# Automatic Multiple-Needle Surgical Planning of Robotic-Assisted Microwave Coagulation in Large Liver Tumor Therapy

**DOI:** 10.1371/journal.pone.0149482

**Published:** 2016-03-16

**Authors:** Shaoli Liu, Zeyang Xia, Jianhua Liu, Jing Xu, He Ren, Tong Lu, Xiangdong Yang

**Affiliations:** 1 School of Mechanical Engineering, Beijing Institute of Technology, Beijing, China; 2 Shenzhen Institutes of Advanced Technology, Chinese Academy of Sciences, ShenZhen, China; 3 Department of Mechanical Engineering, Tsinghua University, Beijing, China; 4 Department of Ultrasound in Medicine, Navy General Hospital of PLA, Beijing, China; 5 Department of Ultrasound, the Chinese PLA General Hospital, Beijing, China; Icahn School of Medicine at Mount Sinai, UNITED STATES

## Abstract

The “robotic-assisted liver tumor coagulation therapy” (RALTCT) system is a promising candidate for large liver tumor treatment in terms of accuracy and speed. A prerequisite for effective therapy is accurate surgical planning. However, it is difficult for the surgeon to perform surgical planning manually due to the difficulties associated with robot-assisted large liver tumor therapy. These main difficulties include the following aspects: (1) multiple needles are needed to destroy the entire tumor, (2) the insertion trajectories of the needles should avoid the ribs, blood vessels, and other tissues and organs in the abdominal cavity, (3) the placement of multiple needles should avoid interference with each other, (4) an inserted needle will cause some deformation of liver, which will result in changes in subsequently inserted needles’ operating environment, and (5) the multiple needle-insertion trajectories should be consistent with the needle-driven robot’s movement characteristics. Thus, an effective multiple-needle surgical planning procedure is needed. To overcome these problems, we present an automatic multiple-needle surgical planning of optimal insertion trajectories to the targets, based on a mathematical description of all relevant structure surfaces. The method determines the analytical expression of boundaries of every needle “collision-free reachable workspace” (CFRW), which are the feasible insertion zones based on several constraints. Then, the optimal needle insertion trajectory within the optimization criteria will be chosen in the needle CFRW automatically. Also, the results can be visualized with our navigation system. In the simulation experiment, three needle-insertion trajectories were obtained successfully. In the *in vitro* experiment, the robot successfully achieved insertion of multiple needles. The proposed automatic multiple-needle surgical planning can improve the efficiency and safety of robot-assisted large liver tumor therapy, significantly reduce the surgeon’s workload, and is especially helpful for an inexperienced surgeon. The methodology should be easy to adapt in other body parts.

## Introduction

Over the past decade, percutaneous microwave (MW) coagulation therapy, as a minimally invasive surgery, has been used extensively in malignant liver tumor treatments due to its safety, rapid recovery, and low cost. In this technique, to destroy an entire malignant liver tumor, two important clinical considerations should be noted: tumor size and accessibility of the lesion. For example, only a single needle is required for a small liver tumor microwave coagulation (MC), whereas multiple overlapping MC needs to be applied to cover irregular and large tumors through a series of single-needle MC [1–3]. Such multiple overlapping MC is more difficult simply due to the placement of multiple needles. Thus, manual treatment planning and execution is dependent on the surgeon’s experience and it is time-consuming.

**Fig 1:**
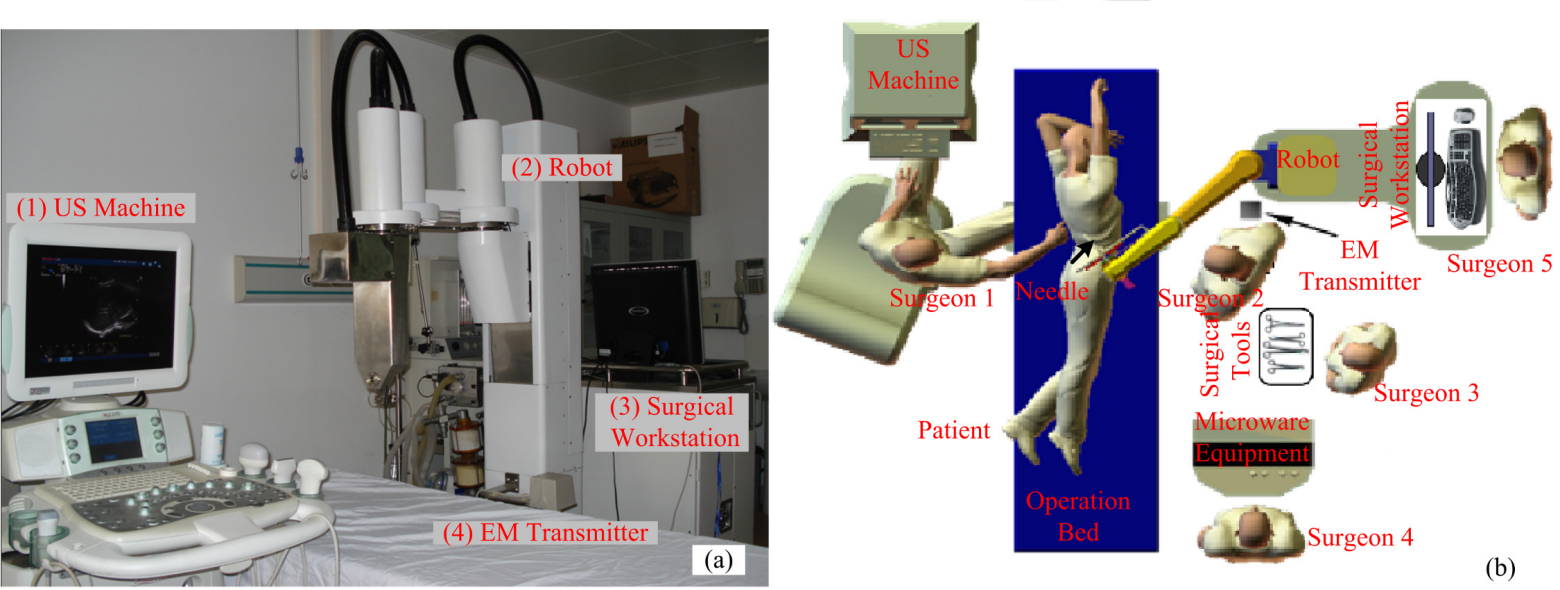
The RALTCT system and the operation environment. (a) The RALTCT system consists of four basic components: (1) the ultrasound (US) machine for two-dimensional (2D) US image display, (2) a five degrees-of-freedom (DOF) robot to manipulate the needle, (3) a PC-based surgical workstation that integrates the surgical navigation software and supervisory control of the robot, and (4) an electro-magnetic (EM) tracking system to record the position and orientation of the US probe. (b) The operation environment (top view).

In this regard, the sophisticated robotic-assisted surgery technique is a candidate for the multiple overlapping MC because of its advantages, such as three-dimensional (3D) visualization, needle placement accuracy, and ease of operation. The RALTCT system, developed by our group, has already been used to treat more than 20 patients with liver or kidney cysts. This demonstrates the feasibility of our robotic system for precise needle placement [4]. The RALTCT system and the surgery environment are shown in Fig 1.

Based on the operation process, we know that preoperative planning is the most fundamental and important step, because it affects all subsequent operations and treatment efficacy. However, existing surgical planning depends mainly on the surgeon’s experience, because the needle insertion trajectory is determined by the surgeon, manually, under the 3D visualization navigation system. However, multiple overlapping MC does not allow us to directly adopt single-needle surgical planning to obtain multiple needle-insertion trajectories because of the difficulties in clinical operation for large liver tumor treatment. For example, (1) a uniform surgical planning framework is expected to be applied to multiple patients, based on the size, shape, and location of the tumor, as well as on the patient’s individual anatomy, (2) the needle insertion trajectory should avoid damaging important organs or tissues (e.g., the ribs or primary blood vessels), and the needle insertion trajectory should avoid interference between needles and minimize damage to healthy tissue, (3) the deformation of soft tissue during needle insertion should be taken into account, and (4) for the manipulability of robot-assisted needle insertion, the planned needle insertion trajectory is restricted within the robot’s reachable workspace; thus, the robot’s movement characteristics must also be considered. For those reasons, surgical planning for multiple needle-insertion of large liver tumor therapy requires development to overcome the abovementioned difficulties and enhance the planning efficiency and quality.

To date, much effort has been applied to promoting surgical planning. The most widely used planning methods are based on virtual reality (VR) and collision detection. In surgical planning, collision detection insures that the surgical tool will not collide with the important tissue of patients. Cristina et al. [5] presented a system called VirSSPA, which generated personalized patients biomodels in VR. Techniques such as image analysis, segmentation, and modeling were used within the system. It can support interventions and the training of surgeons. Butnariu et al. [6] presented a pre-planning stage based on a VR technique to generate and optimize the needle trajectory of the robotic brachytherapy procedure. Schumann et al. [7] presented a visualization method that highlighted less-suited trajectories and projected a risk structure map directly on the 2D slices. This visualization helps the surgeon to directly select a second point on the linear access trajectory in the 2D image slices (the user-defined target point first). Shamir et al. [8] presented a preoperative planning method to assist the neurosurgeon in selecting the safest trajectory. The method presents multiple candidate trajectories on the outer head surface. The surgeon can select or revise the trajectory using interactive 3D visualization and obtain a risk measure of the trajectory to critical brain structures. Although these methods show some benefits for the surgeon in deciding the trajectories of tools, they are still heavily dependent on the surgeon’s skills and the optimal surgical planning is not developed. For this reason, some researchers introduce force feedback haptic device in surgical planning. For example, Villard et al. [9] presented a 3D simulator that used VR and haptic devices to make simulations and training realistic and effective. Optimal placement of not only a single needle but also of several optimal placements of a needle sequentially for large tumor treatment was developed by volume minimization. However, Seitel [10] pointed out that the method did not give as good results as expected because of the very long execution time and the difficulty in determining the optimal parameters for each new tumor shape in individual patients. Moreover, to simplify the several optimal needle placements for large tumor treatment, the method assumes that several insertions are performed sequentially with a needle and each treatment burns a different area, one by one. Thus, there is no interaction between the different parts of the process. However, in multiple overlapping MC, challenges such as the interaction between different needles should be considered. Thus, those types of surgical planning are primarily suited to relatively simple surgeries, such as neurosurgery and dental surgery, because the structures in the brain and oral cavity can be considered more rigid. Also such surgical planning can be adopted for single-needle insertion in the abdomen to obtain feasible insertion trajectories.

To reduce the dependence on surgeon’s skill, the concept of ‘virtual fixture’ (VF) [11, 12] has been adopted in surgical planning. Generally, the method establishes VF by a force control to prevent the robot from moving in prohibited areas [13] or guide medical tools to move in the desired direction [14]. Recently, this method was extended to multiple manipulators by an example of a surgical knot placed at a target point [15]. The extended method provides spatial and temporal guidance for multiple manipulators within the same workspace (such as with the *Steady Hand* [16] and *da Vinci* systems [17]). The VF-based method still allows the surgeon to control the robot ultimately. Based on preoperative surgical planning, the surgeon can also adjust the surgical plan by the actual intraoperative situation. The VF-based method improves operation safety and reduces the surgeon’s planning workload. However, it does not meet the requirements for abdominal surgery because many empirical coefficients to establish VF must be identified and the operation force will cause changes in the operation environment.

Automatic or semi-automatic surgical planning is more suitable for multiple overlapping MC, because the interaction between several needles (or multiple manipulators of the robot) and the deformation of soft tissue during insertion should be considered. These methods focus on appropriate empirical models and reasonable optimizing criteria and rarely depend on the surgeon’s skill. Thus, automatic or semi-automatic surgical planning methods are suggested for the multiple manipulators in the robotic-assisted surgery systems. Spencer et al. [18] presented a method to provide tools trajectories within a compact surgical workspace, based on the anatomical structure of the chest, collision avoidance, and multiple-objective optimality criteria. Azimian et al. [19] extended the work to achieve implementation for target reachability and a dexterity assessment of the instrument. In this research, optimal placement of the *da Vinci* manipulators was determined by multiple-objective optimality criteria. Also, the kinematic and geometric requirements of the problem were incorporated into the constraints. Although these methods are quite efficient and relatively independent of the surgeon’s skill, they may be difficult to apply to other types of surgery because the effectiveness of planning results depend mainly on complete optimization criteria and special physiological structures such as chest. Thus, due to the absence of a physiological structure as a reference for the candidate entry points in the abdominal environment, these methods cannot be applied directly to multiple overlapping MC. Moreover, the number of the planning trajectories in these robotic-assisted surgery systems is invariable, whereas the number of required planning trajectories varies with liver tumor size. For robot-assisted multiple overlapping MC for large tumor treatment, the depth of insertion, ablation of healthy tissue, and avoidance of risk-structure insertion are important considerations in the optimizing criteria. Baegert [20, 21] and Villard [22] presented an automatic insertion trajectories planning method. The insertion trajectory is the line that connects the given target in the tumor and an insertion point on the skin. The insertion zones on the skin are determined by hard constraints, which means that the trajectories in the zones will not hit a critical structure or exceed the needle length. Then, based on soft constraints, such as the distance to critical structures, the trajectories allowed according to the hard constraints are then rated. Several soft constraints are combined by a weighted sum to obtain an overall rating of a given needle insertion trajectory. However, some researchers have pointed out that the weighted sum rating may be misleading. The weight of the constraints should be considered carefully to avoid a potential complication at a critical structure. Because all of the calculations are based on a surface mesh, the precision of proposed algorithms is decided by the discrete size of the surface. Seitel et al. [10] extended the work of Baegert and Villard to achieve rapid and robust implementation for automatic or semi-automatic trajectory planning. Also, the possible insertion zones are determined based on hard constraints. Then, the quality of insertion trajectories in the zones is rated by soft constraints. The concept of Pareto optimality has been proposed to allow for a weight-independent proposal of insertion trajectories. The planning method can detect unsafe, and propose safe, insertion trajectories for the interventional radiologist. Because the planning results are based on the mesh points of the critical structures, the resolution of the mesh should be carefully chosen to balance runtime and planning accuracy. Also, the constraints directly computed based on graphics rendering are limited by the resolution of the screen used. Schuman et al. [23] presents a planning approach independent of the mesh representation of the critical structures. The cylindrical projection with the center at the target point in the tumor is used to compute the constraint maps for each restriction. Then, the optimal insertion trajectories can be obtained by a constraint-specific rating function and a weighted combination of all constraints. Although the total computation time for determining the optimal insertion trajectory is short, the weight factors of each constraint must still be set manually. Moreover, this planning method does not include an ability to solve multiple-needle insertion trajectories. Wong et al. [24] developed a method that can perform optimization and re-planning of multiple ablation probe placements while respecting clinical needs, such as avoiding ablation of critical structures, reducing ablation of healthy tissue, and overlapping of ablation zones. The ablation probe placement is formulated as an optimization problem with hard and soft constraints. Then, a mixed variable mesh adaptive direct search is used to yield a feasible solution within a few iterations. However, the interaction between the different insertion probes is not considered, because the assumption is that the probe will not be pulled completely out of the liver and redirected to a new location. Chen et al. [25] presented a preoperative protocol using a regular prism and a regular polyhedron model to minimize the number of ablation spheres, optimize the overlapping mode, and determine the electrode placement. Mundeleer et al. [26] presented an algorithm to optimize the set of destruction centers with common spherical assumptions that was completely parametrical. The main advantages are the ability to generalize the scheme to any tumor size and shape. However, trajectory planning was not developed. Yang et al. [27] developed a robotic ablation system and suggested a voxel-growing algorithm for large liver tumors. The planned target points were capable of destroying the entire liver tumor using the fewest needles. Similarly, optimal multiple-needle insertion trajectories were not addressed. Ren et al. [28] developed a semi-automatic planning and guidance method for multiple overlapping RFAs. Multiple-objective optimization, including both clinical and technical constraints, was used. However, at the beginning, the clinician must specify a set of entry points manually. Thus, the optimal results are local optima. Moreover, these methods did not consider the deformation of soft tissue during the planning.

To sum up, we further extended the problem in this paper to develop a clinically accurate surgical planning system, particularly for multiple overlapping MC. The proposed method incorporated constraints that are encountered in clinical practice, but which have not been fully addressed previously, including 1) deformation of soft tissue during needle insertion, and 2) interactive constraints between multiple needles. To identify the optimal multiple insertion trajectories, in this paper, the planning goals are translated into a mathematical problem, which can then be solved by numerical techniques. The main contributions of our work are twofold. First, we translated multiple trajectories planning into a mathematical model to accurately solve the feasible insertion zones, which are termed needle CFRW in this paper, by an analytical expression. The method is independent of the mesh representation of related structures and the resolution of the screen used. Second, we analyzed constraints, including not only the clinical constraints but also operational constraints of the robot for automatic multiple trajectories planning to promote the development of robot-assisted large tumor treatment. The automatic surgical planning method aims to be helpful especially for the inexperienced surgeon and reduce the surgeon’s workload.

The remainder of this paper is organized as follows: First, we describe the procedures of robot-assisted multiple overlapping MC and constraints for larger liver tumors. Then, the analytical expression of needle CFRW with constraints of multiple overlapping MC is solved to evaluate insertion safety. The optimal needle-insertion trajectory, which must be within the corresponding needle CFRW is obtained. Finally, a simulation of multiple needle trajectory planning and the *in vitro* experiment with multi-needle insertions are shown and discussed.

## Materials and Methods

In the manuscript, the approval for the surgery environment of the *in vitro* experiment for a pig liver is not necessary. Because in China, pig liver is a kind of common food. We got it from the Zhaolanyuan supermarket in Tsinghua University, Beijing, China. Also, the pig liver *in vitro* experiment used in this study have been described in Ph.D.thesis of Shaoli Liu. Research on Multi-Needle Surgical Planning of Robot-assisted microwave coagulation in liver cancer therapy [D]. Beijing, Department of Precision Instruments and Mechanology, Tsinghua University, 2012

The liver in the new experiment was gotten from the SPF level animal laboratory of the animal laboratory center in Navy general hospital of PLA. And it was carried out under an approved animal protocol of the Guide for the Care and Use of Laboratory Animals. The name is Institutional Animal Care and Use Committee of Navy General Hospital of PLA.

### Procedures of robot-assisted multiple overlapping coagulation for larger liver tumors

**Fig 2:**
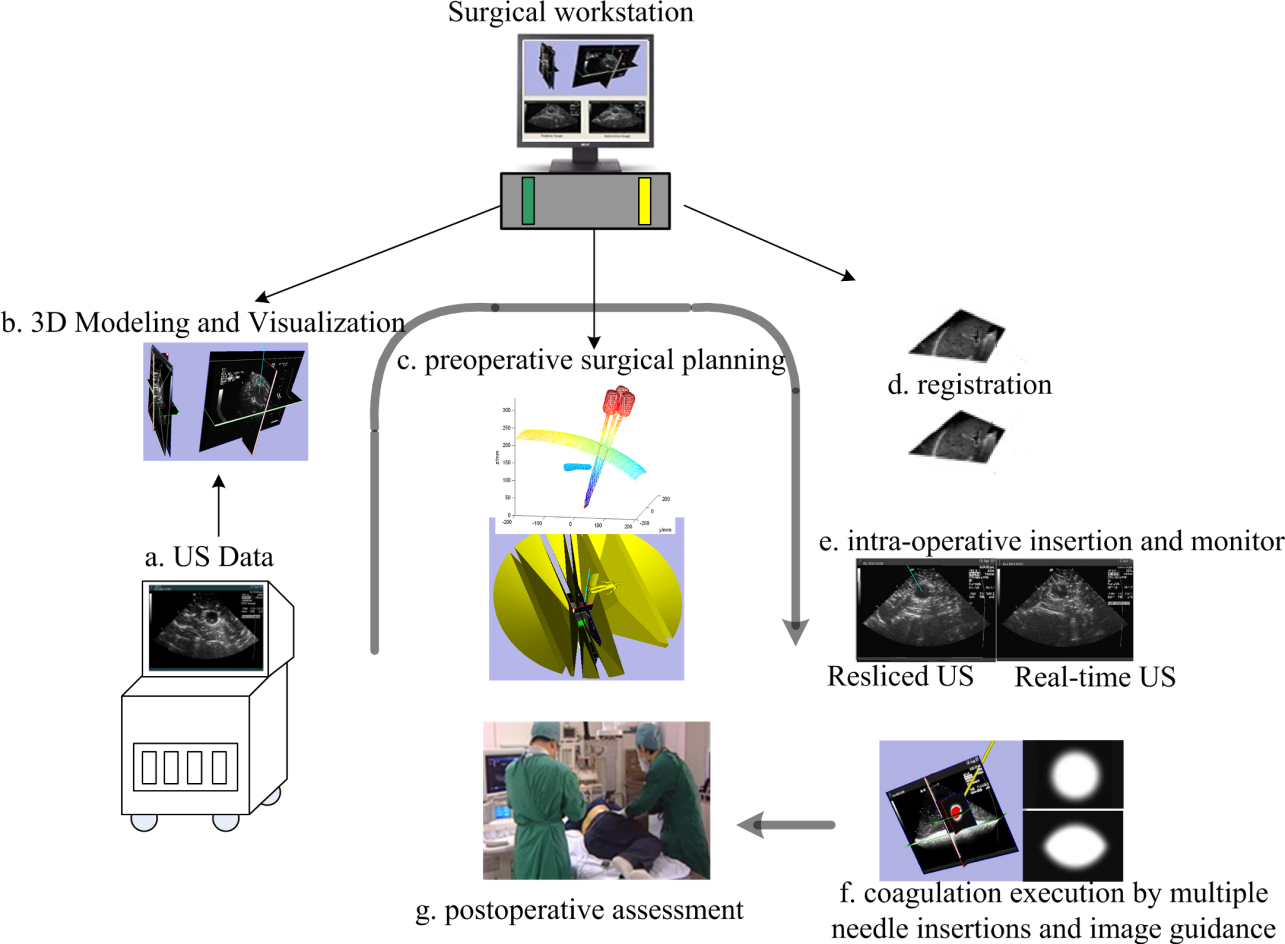
Overall schematic of a planning and US image-based navigation system for large liver tumors. a-c are parts of step (1), d-f are parts of step (2), g is part of step (3).

The traditional clinical multiple overlapping MC therapy for large liver tumor usually consisted of several steps. In *step 1*, the surgeon obtained a two-dimensional (2D) ultrasound (US) image of the tumor by US probe scanning. Then, based on the size and position of the tumor, the surgeon had to determine the configured microwave energy, action time, and the number of required needles. In *step 2*, the surgeon determined the insertion scheme of multiple needles, including the positions of the destruction targets in the tumor and the corresponding entry points on the skin, based on experience. In *step 3*, after local anesthesia, the surgeon manually placed the needles to the desired locations with a needle-guiding device attached to the US probe, one by one. The needle-guiding device ensures that the needle insertion trajectory can always be tracked with the real-time US image to reduce the danger of surgery. Once all needles are placed at the predetermined target sites, the needles can then generate thermal energy to destroy the tumor cells [2]. Based on the procedures of the traditional clinical multiple overlapping MC therapy for large liver tumors and the typical operating procedures of the RALTCT system using a single needle for liver or kidney cysts [4], this paper proposes typical operation procedures for the RALTCT system using multiple needles for large liver tumors. The procedure consists of three steps: (1) preoperative off-line surgical planning, (2) intraoperative therapy, and (3) postoperative assessment. The details are described below and shown in Fig 2. The individual in this manuscript has given written informed consent (as outlined in PLOS consent form) to publish these case details.

Step (1): Preoperative off-line surgical planning. The surgeon can obtain a 3D model of the liver including the tumors and the blood vessels reconstructed from a group of scanning 2D US images [4]. Also, the point cloud positions for ribs and the patient’s abdominal epidermis can be obtained using an electro-magnetic (EM) tracking device, such as the *Ascension Bird* position sensor. Then, the number of needles and the corresponding positions of targets in the tumor can be determined. Based on multiple-needle surgical planning, the optimal needle-insertion trajectories and the corresponding entry points on the patient’s abdominal epidermis can be determined.

Step (2): Intraoperative therapy. The preoperative specific liver model and needle-insertion trajectories are registered in an intraoperative surgical model of the patient based on vessels of the liver [4]. Then, the needle-guiding device, which is fixed at the end of the robot, will be manipulated robotically to the desired position. The surgeon then manually inserts the needle to hit the target under the guidance of US images. Following placement of one needle in the desired position, the operation is repeated until all needles have been inserted and reached their targets. Then, the surgeon can switch on the MW machine to perform the thermal MC. The needle-targeting accuracy can be further improved by fine adjustment of the needle-insertion process with real-time feedback from the onscreen display.

Step (3): Postoperative assessment. After the operation, the surgeon assembles a series of 2D US images of the liver and surgery information is collected to confirm that the surgery plan was successfully executed and to evaluate the effectiveness of the operation.

### Goals, constraints, and procedures of multiple-needle surgical planning for the medical robot

The purpose of multiple-needle surgical planning is to determine the optimal needle insertion trajectory to ensure that the needle accurately reaches its target so as to destroy the liver tumor completely. Because the needle has sufficient rigidity, its insertion trajectory can be described as a line connecting the entry point on the patient’s abdominal epidermis with the corresponding target in the tumor. Thus, this paper proposes that multiple-needle surgical planning will facilitate automatic determination of the entry point corresponding to the optimal insertion trajectory of each needle. In addition, based on the existing studies we know that the optimum number of needles and the corresponding targets in the tumor is also a main factor affecting the treatment results. Since this is a topic of great research interest. It is, however, independent of our trajectory planning objective. Once a reliable mathematical model to accurately estimate the number of needles and corresponding targets in the tumor [25, 29–30] is available, the targets position can be easily integrated into our method. The characteristics of the surgery environment and the constraints on surgical planning are discussed in the following paragraphs.

**Fig 3:**
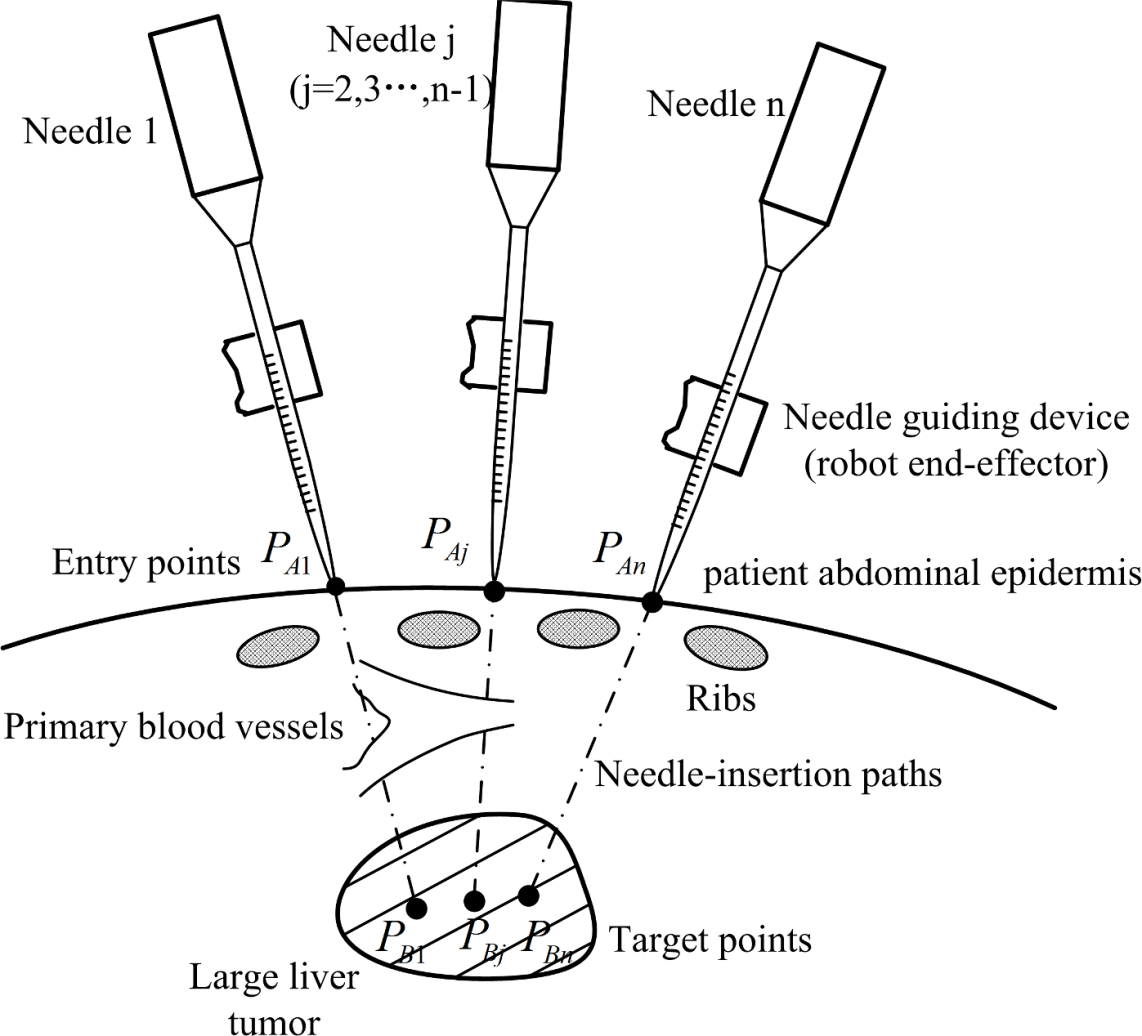
Schematic diagram of the environment of an abdominal operation.

The most important characteristic is the complexity of the operating environment of the abdominal cavity. This is a common characteristic of abdominal cavity surgery. As shown in Fig 3, the human abdominal cavity is a complex 3D environment containing many important tissues and organs, such as the ribs and blood vessels. Additionally, the number, shape, and position of these organs vary among patients. Furthermore, robot-assisted multiple-needle surgery for larger liver tumors also has several typical characteristics, such as 1) the variability in the volume of the liver tumor among patients. Thus, the number of needles needed to destroy the entire tumor is also variable. Also, the task of surgical planning differs among individual patients. 2) According to the proposed procedures for robot-assisted multiple-needle surgery, the needles are inserted one by one. The motion associated with needle insertion will cause deformation of the liver. Thus, the positions of targets and the shapes of blood vessels differ following needle insertion. Also, the inserted needle represents a new obstacle that must be avoided by the trajectory of insertion of subsequent needles. Thus, the planning environment for each needle is different.

Based on these characteristics, the constraints for the needle-insertion trajectory include the following. 1) The needle insertion trajectory should avoid interfering with the obstacles in the abdominal operation environment, such as blood vessels, ribs and previously inserted needles. Because unavoidable deviation from a planned trajectory in practice may cause a fatal injury, needle-insertion trajectories that are very close to vital organs should be abandoned. 2) To destroy the entire tumor with the minimum damage and decrease the recurrence rate, the tip of the needle should reach the corresponding target accurately. Thus, the displacement of targets caused by the deformation of the liver should be considered, as should 3) the length of the needle, and 4) the movement capabilities of the robot.

As shown in Fig 3, the line segments between the entry points *P*_*Ai*_ (*i* = 1,2⋯,*n*,*n* ∈ *N*^+^) on the patient’s abdominal epidermis and the corresponding target points *P*_*Bi*_ (*i* = 1,2⋯,*n*,*n* ∈ *N*^+^) in the large liver tumor are the multiple-needle-insertion trajectories, and the targets are specified and fixed during surgical planning. In this case, to identify the corresponding needle insertion trajectory, the parameters to be planned are only the entry points *P*_*Ai*_. Based on this condition, the following functions of the multiple-needle surgical planning are proposed: 1) The modeling of important tissues, such as ribs and blood vessels in the abdominal cavity, needles, and the abdominal epidermis of the patient. The model provides constraints for the planning. 2) A deformation analysis of the liver. The purpose is to determine the displacements of the targets and the blood vessels caused by needle insertion. Then, the information for the planning environment will be revised for planning of the subsequent needle insertion trajectory. This function can improve the efficacy and safety of the surgery. 3) Determining the set of all feasible needle-insertion trajectories for each needle. With the help of the set, even an inexperienced surgeon can choose or adjust a feasible needle insertion trajectory quickly. Also the optimal needle-insertion trajectory can be obtained automatically within the set with optimization criteria. Thus, this paper proposes that the multiple-needle surgical planning should be based on the needle collision-free reachable workspace (CFRW). The needle CFRW is a set of needle insertion trajectories that the needle can reach with no collision between the needle and obstacles. 4) Proposing optimization criteria for the needle-insertion trajectory. The optimal needle-insertion trajectory should be within the needle CFRW. Finally, the procedures of multiple-needle surgical planning should adjust to the characteristics of multiple-needle surgery, such as including each inserted needle as a new obstacle and adapting the plan for different numbers of needles for different patients.

**Fig 4:**
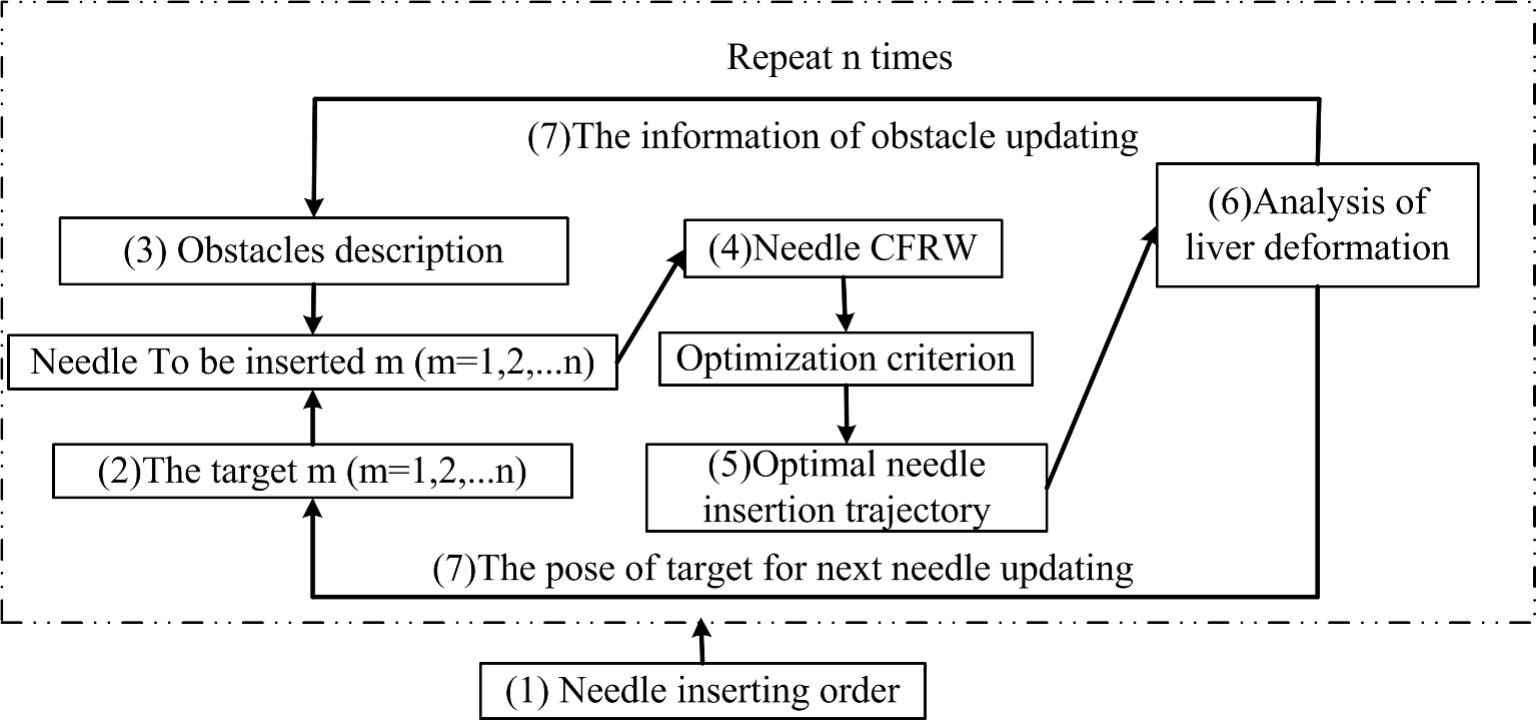
Flowchart of multiple-needle insertion trajectory planning (the obstacles in the first needle insertion trajectory planning environment are the ribs and blood vessels in the abdominal cavity. The obstacles in the subsequent needle-insertion-trajectory planning environment include the previously inserted needles as new obstacles).

The procedures of multiple-needle insertion trajectory planning can be divided into the following steps, as shown in Fig 4:

1. determine the order of needle insertion,
2. determine the insertion targets,
3. describe the obstacles in the abdominal operation environment,
4. calculate the needle CFRW, based on the order of needle insertion,
5. select the optimal needle insertion trajectory,
6. analyze the deformation of the liver,
7. update the information on obstacles and target corresponding to the next needle, and
8. repeat steps (3)-(7) until all required needles have been planned.

For the subsequent needle-insertion trajectory planning, the information on obstacles is updated.

### Optimal multiple-needle insertion trajectory planning

In this section, our mission is to obtain the optimal multiple-needle insertion trajectories. The basic principle is to compute the feasible insertion set for each needle with the above constraints, from which the global optimal needle-insertion trajectory is extracted, according to the proposed optimization criterion. To this end, we introduced the needle CFRW, in which no interference occurs between the inserted needles and the critical structures. Based on our method, the clinical constraints and rating factors—such as the safety margin around target constraints, tangency constraints, needle length constraints, the distances to critical structures, and trajectory length—can be expressed as parameters in the non-linear equations that describe the needle CFRW. The prerequisites include segmentation of the important organs and the implicit expression of these organs’ surfaces. When the deformation of soft tissue is considered, the segmentation is carried out based on the deformed liver model [31]. The proposed multiple-needle insertion trajectory planning is a general method regardless of individual operation environment differences. The needle-insertion trajectory planning includes two parts: the calculation of the boundary of the needle CFRW and the selection of the optimal needle insertion trajectory.

#### Analytical description of needle CFRW boundary in a multiple-needle environment

As shown in Fig 3, there are many obstacles (e.g., primary blood vessels, ribs, and inserted needles) in the abdominal operation environment. To simplify the computation, for each needle, the CFRW with several obstacles is regarded as the intersection of the CFRWs for every obstacle.

For the needle j (j = 2, 3, …, n) which is the j^th^ inserted needle, the previously inserted needles can be considered as obstacles (Fig 3). Assuming that needle 1 to needle (j-1) have all been inserted accurately along the planned needle insertion trajectory, in this section, the process to solve the CFRW of needle j will be described in detail.

**Fig 5:**
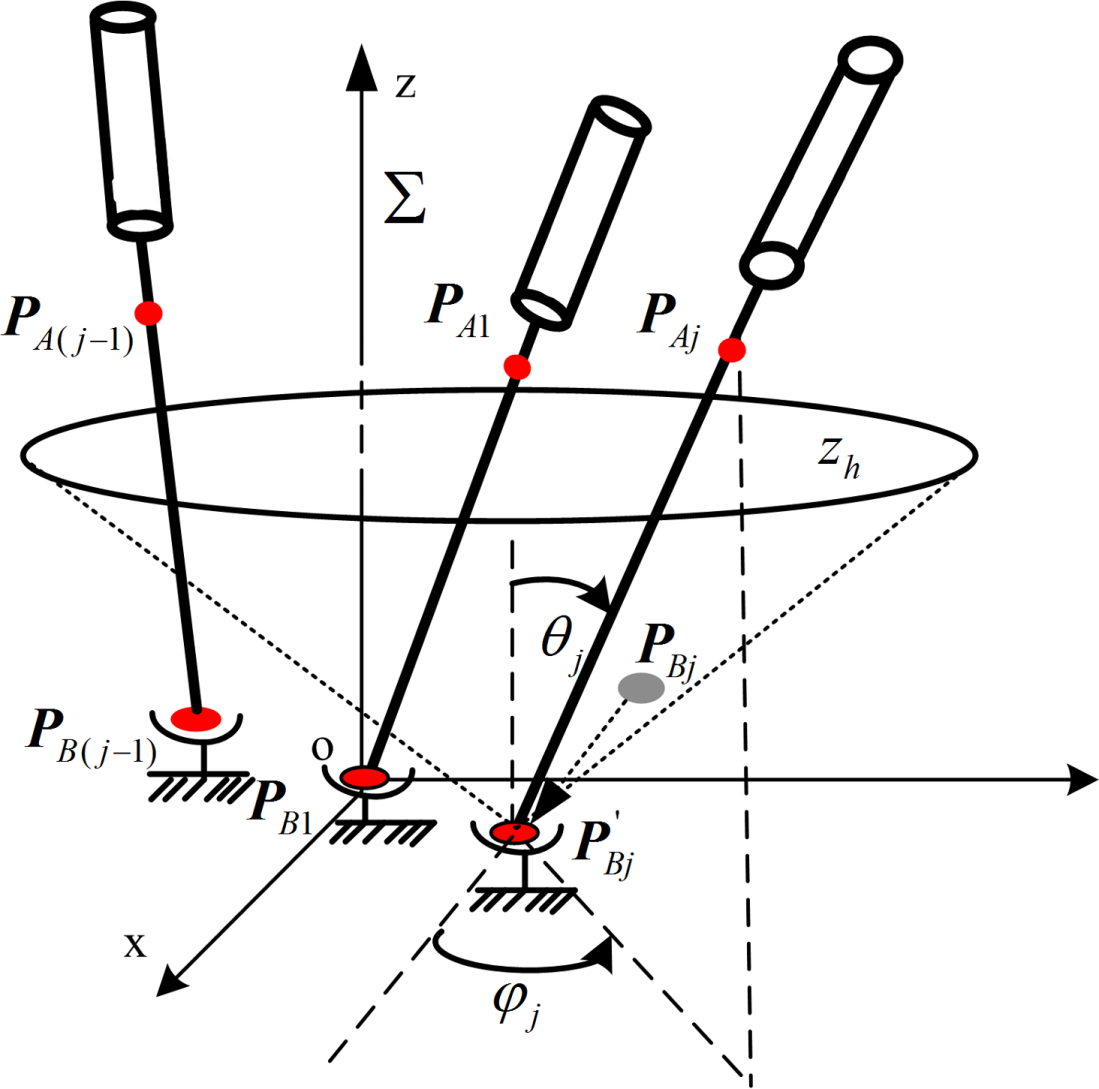
Needle-insertion trajectory planning for needle j.

As shown in Fig 5, *P*_*B*1_ is the target point corresponding to needle 1 and ***P***_A1_ is the optimal entry point; *P*_*B*(*j*−1)_ is the target point corresponding to needle (j-1) and ***P***_A(j-1)_ is the optimal entry point. For the planning of needle j’s insertion trajectory, the inserted needles 1 to (j-1) are new obstacles. The needle-insertion trajectory for needle j can be specified by the target point *P*_*Bj*_ and entry point *P*_*Aj*_. Because *P*_*Bj*_ is known and it is the desired location of the tip of the needle should be arrived accurately, we consider *P*_*Bj*_ to be fixed during the planning and the *P*_*Aj*_ to be variable. Then the needle j can be simplified as an arm with a single ball-hinge joint, the center of which is *P*_*Bj*_. Similarly, the previous needle-insertion trajectories for needles 1 to (j-1) are also simplified as an arm with a single ball hinge joint.

To solve the needle CFRW, we first established the world coordinate frame Σ on needle 1, as shown in Fig 5, where the z-axis is vertical-up and the origin coincides with *P*_*B*1_. To unify the coordinates, all needle-insertion trajectories are planned in Σ. According to ***P***_A(*j*−1)_ = [*x*_*j*−1_, *y*_*j*−1_, *z*_*j*−1_]^T^ ∈ *R*^3^ and ***P***_B(*j*−1)_ = [*x*_o(*j*−1)_, *y*_o(*j*−1)_, *z*_o(*j*−1)_]^T^ ∈ *R*^3^ which have been already planned, the position of the inserted needle (j-1) can be solved.

In Σ, the end of needle j’s output variable is ***P***_A*j*_ = [*x*_A*j*_, *y*_A*j*_, *z*_A*j*_]^T^ ∈ *R*^3^, and ***P***_B*j*_ = [*x*_o*j*_, *y*_o*j*_, *z*_o*j*_]^T^ ∈ *R*^3^ is a constant value. Assuming the displacement of ***P***_B*j*_ caused by the deformation of liver is ***δ***_*j*_ = [*δ*_x*j*_, *δ*_y*j*_, *δ*_z*j*_]^T^, the actual target of the tip of needle (j-1) is $P_{\text{B}j}^{\prime }=P_{\text{B}j}+\delta_{j}={\left[x_{\text{o}j}+\delta_{\text{x}j},y_{\text{o}j}+\delta_{\text{y}j},z_{\text{o}j}+\delta_{\text{z}j}\right]}^{T}$ as shown in Fig 5. Then, we set up the model of needle j (Fig 5), which is an arm with a single ball-hinge joint, to coincide with $P_{\text{B}j}^{\prime }$. Because the needle’s rotation around its own axis would not affect the position of the needle, the needle position can be represented by the other two rotational DOF, which are the rotation angles *θ*_*j*_, *φ*_*j*_ around the *y*-axis and the *z*-axis in Σ.

To simplify the computation, the desired boundary of CFRW for needle j, which is a 3D curved surface is decomposed into layers along the*z*-axis direction in Σ. In this case, the boundary is a one-dimensional (1D) curve in each 2D layered *xy*-plane. Then, let the *xy*-plane be recorded as *z*_*h*_-plane with *z* = *h*, *h* ∈ [0, *h*_max_] (according to our clinical experience, *h*_max_ = 200*mm* in our RALTCT system). Then, the boundary of CFRW for the needle j will be solved in each *z*_*h*_-plane. In the *z*_*h*_-plane, the generalized coordinates ***q*** = [*x*_*j*_, *y*_*j*_, *θ*_*j*_, *φ*_*j*_] ∈ *R*^4^ are used to describe the position and orientation of needle j, where *x*_*j*_, *y*_*j*_ are the output coordinates of the intersection point of needle j and the *z*_*h*_-plane. Then, in the *z*_*h*_-plane, the kinematic constraint equation of needle j in Σ is denoted as:

$$\Phi (q)=\left[\begin{array}{c} x_{j}-(h-(z_{\text{o}j}+\delta_{\text{z}j}))\times \text{tan}\theta_{j}\times \text{cos}\varphi_{j}-x_{\text{o}j}-\delta_{\text{x}j}\\ y_{j}-(h-(z_{\text{o}j}+\delta_{\text{z}j}))\times \text{tan}\theta_{j}\times \text{sin}\varphi_{j}-y_{\text{o}j}-\delta_{\text{y}j} \end{array}\right]=0$$ (1)

where *x*_*j*_, *y*_*j*_, *θ*_*j*_, and *φ*_*j*_ are variables, and *x*_o*j*_, *y*_o*j*_, *z*_o*j*_, *δ*_x*j*_, *δ*_y*j*_, *δ*_z*j*_, and *h* are constants.

For the safety and manipulability of the operation, the angles *θ*_*j*_ and *φ*_*j*_ in the RALTCT system are restricted, as follows:

$$\begin{array}{l} \theta_{\text{min}}\leq \theta_{j}\leq \theta_{\text{max}}\\ \varphi_{\text{min}}\leq \varphi_{j}\leq \varphi_{\text{max}} \end{array}$$ (2)

The limits of the two parameters are set based on clinical constraints and robot movement constraints. For example, there is always a safety margin around the target. Also, the trajectory should be shorter than the length of the needle to be inserted in the clinical surgery. Then, based on the patient’s US image and the 3D reconstructed model of the liver, the constraints can be translated into the limits of these two parameters by geometrical relationships. Based on the robot structure, we know that the insertion angle, which is decided by the two parameters, is also limited by joints four and five of the robot [4]. Through the robot’s kinematics, the limits of the two parameters can be calculated. Then, the final parameters, *θ*_min_, *θ*_max_, *φ*_min_, and *φ*_max_ will be decided by integrating all of the constraints. Eq 2 can then be rewritten by replacing the constrained variables *θ*_*j*_ and *φ*_*j*_ with unrestrained variables *w*_1_ and *w*_2_:

$$\begin{array}{l} \theta_{j}=\frac{1}{2}(\theta_{\text{max}}+\theta_{\text{min}})+\frac{1}{2}(\theta_{\text{max}}-\theta_{\text{min}})\;\text{sin}\;w_{1}\\ \varphi_{j}=\frac{1}{2}(\varphi_{\text{max}}+\varphi_{\text{min}})+\frac{1}{2}(\varphi_{\text{max}}-\varphi_{\text{min}})\;\text{sin}\;w_{1} \end{array}$$ (3)

Next, the collision-free equation between the needle j and obstacles needs to be established. To this end, the mathematical descriptions of needle j and needle (j-1) are presented.

**Fig 6:**
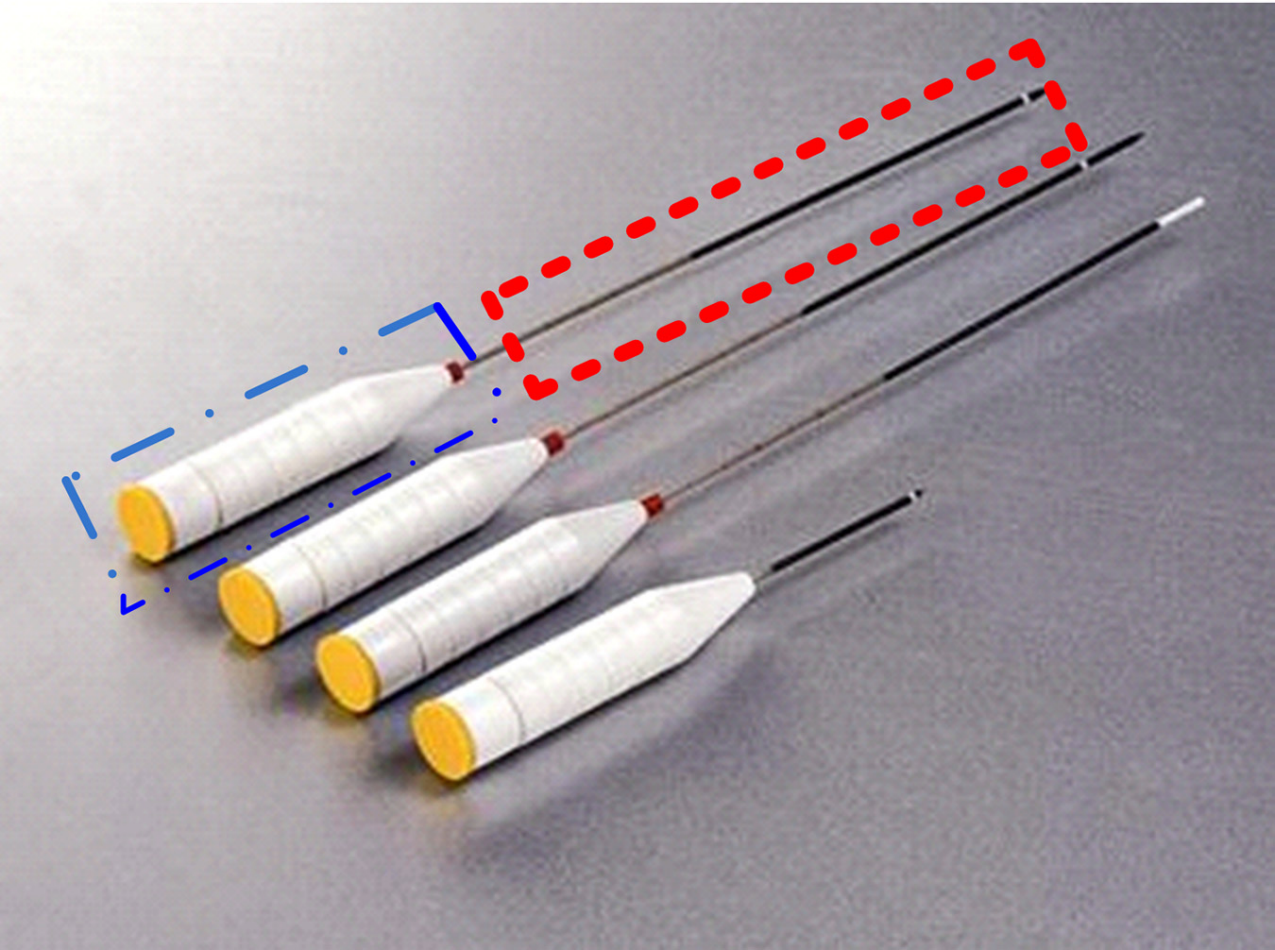
Stereogram of the needle.

As shown in Fig 6, the needle consists of two parts: the needle-tube (within the dot dash line) and the needle-head (within the dotted line). For safety, there must be no collision between every parts of the two needles. In this case, the two parts are represented by mathematical descriptions.

**Fig 7:**
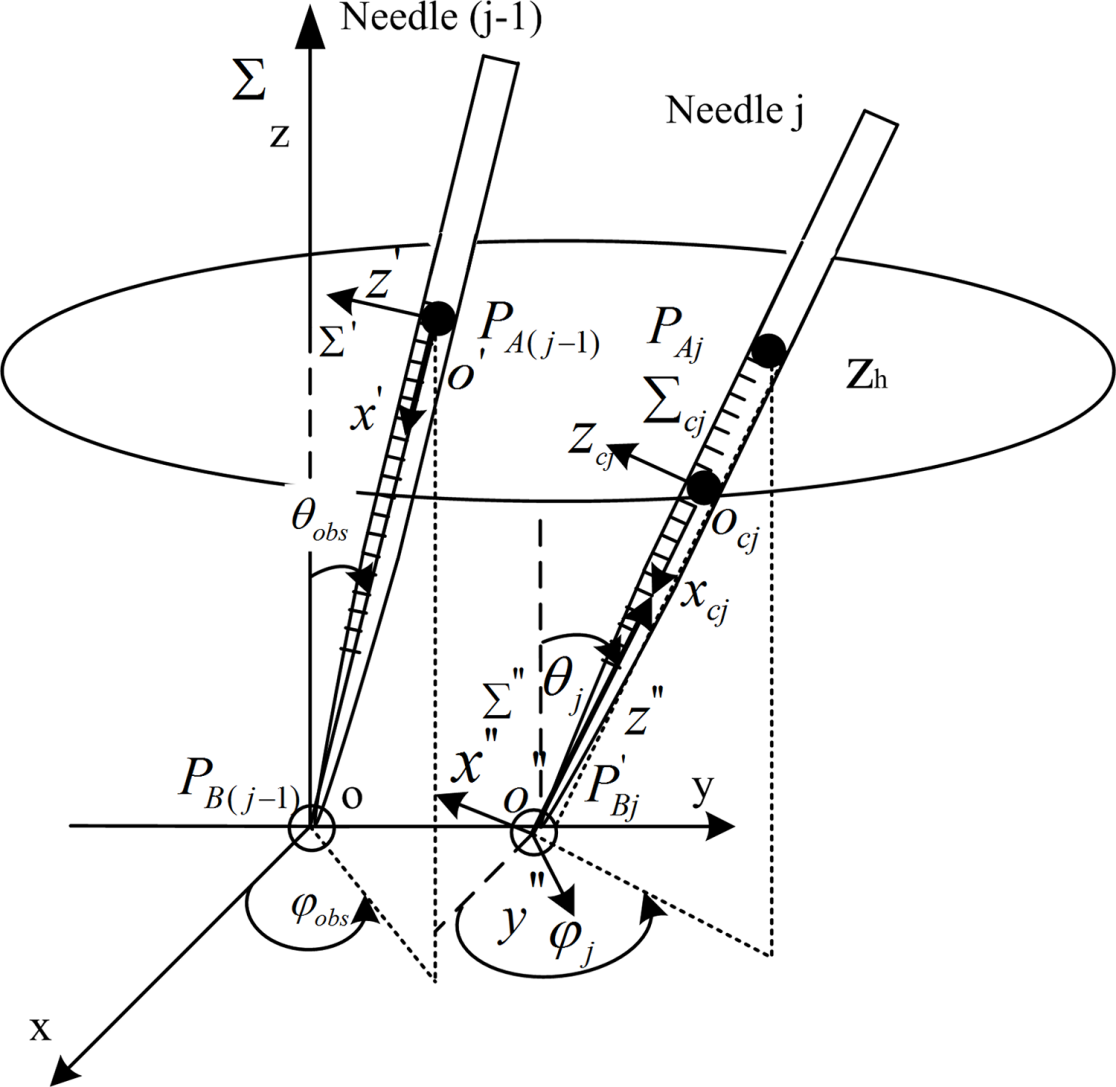
The coordinate frames.

We attach a local coordinate system Σ′ at the geometric center of the needle-head, as shown in Fig 7, and the *x*′-axis coincides with the axis of the needle-head, while the *z*′-axis is perpendicular to it.

Then, in Σ', the curved surface of the needle-head for needle (j-1) can be described by a super-quadric [32, 33] with a canonical form as follows:

$$f_{{\text{n}}_{\left(j-1\right)}}^{\prime }({\left[a_{x}^{\prime },a_{y}^{\prime },a_{z}^{\prime }\right]}^{\text{T}})={\left(a_{x}^{\prime }/60\right)}^{8}+{\left(a_{y}^{\prime }/0.1\right)}^{2}+{\left(a_{z}^{\prime }/0.1\right)}^{2}-1000=0$$ (4)

where ${\left[a_{x}^{\prime },a_{y}^{\prime },a_{z}^{\prime }\right]}^{T}\in R^{3}$ is the point coordinate on the curved surface of the needle-head for needle (j-1) in Σ'. Because the shape and size of every needle is the same, the super-quadric with canonical form for each needle-head can all be written as Eq 4. However, the positions of the inserted needles differ in world coordinate frame Σ, so the equation that describes the needle-head of the different inserted needles in Σ is related to its position.

Fig 7 shows the relationship between Σ' and Σ. Let ***T***_*obs*_ be the homogeneous transformation matrix from Σ' to Σ. Then, point coordinates [*a*_*x*_, *a*_*y*_, *a*_*z*_]^T^ in Σ can be converted by ${\left[a_{x}^{'},a_{y}^{'},a_{z}^{'}\right]}^{\text{T}}$ in Σ':

$${\left[a_{x},a_{y},a_{z}\right]}^{\text{T}}=T_{obs}{\left[a_{x}^{'},a_{y}^{'},a_{z}^{'}\right]}^{\text{T}}$$ (5)

where ***T***_*obs*_ can be represented based on the robot’s kinematics:

$$T_{obs}=\left[\begin{array}{cccc} 1 & 0 & 0 & x_{o'}\\ 0 & 1 & 0 & y_{o'}\\ 0 & 0 & 1 & z_{o'}\\ 0 & 0 & 0 & 1 \end{array}\right]\left[\begin{array}{cccc} \text{cos}\;\varphi_{obs} & -\text{sin}\;\varphi_{obs} & 0 & 0\\ \text{sin}\;\varphi_{obs} & \text{cos}\;\varphi_{obs} & 0 & 0\\ 0 & 0 & 1 & 0\\ 0 & 0 & 0 & 1 \end{array}\right]\left[\begin{array}{cccc} \text{cos}\;\theta_{obs} & 0 & \text{sin}\;\theta_{obs} & 0\\ 0 & 1 & 0 & 0\\ -\text{sin}\;\theta_{obs} & 0 & \text{cos}\;\theta_{obs} & 0\\ 0 & 0 & 0 & 1 \end{array}\right]\left[\begin{array}{cccc} \text{cos}(\pi /2) & 0 & \text{sin}(\pi /2) & 0\\ 0 & 1 & 0 & 0\\ -\text{sin}(\pi /2) & 0 & \text{cos}(\pi /2) & 0\\ 0 & 0 & 0 & 1 \end{array}\right]$$ (6)

Because the length *l* of the needle-head is a constant, the coordinate of origin ***o***' in Σ can be written as:

o'=[xo',yo',zo']T=[l2×sinθobs×cosφobs+xo(j−1),l2×sinθobs×sinφobs+yo(j−1),l2×cosθobs+zo(j−1)]T (7)

where, *φ*_obs_ = arctan((*y*_*j*−1_− *y*_o(*j*−1)_) / (*x*_*j*−1_− *x*_o(*j*−1)_)), $\theta_{\text{obs}}=\text{arct}\;\text{a}\;\text{n}(\sqrt{{\left(x_{j-1}-x_{\text{o}(j-1)}\right)}^{2}+{\left(y_{j-1}-y_{\text{o}(j-1)}\right)}^{2}}\;/\;(z_{j-1}-z_{\text{o}(j-1)}))$.

According to Eqs 4, 5, 6 and 7, the description function *f*_*needle*(*j*−1)_ of the curved surface of the needle-head in Σ can be obtained.

Similarly, the other obstacles (*e*.*g*., needle-tubes, ribs, and primary blood vessels) can also be uniformly represented as *f*_*obs*_ in Σ using the third-order continuously differentiable super-quadric.

Then, the mathematical description of the curved surface of needle j has to be presented. The super-quadric with canonical form that described the needle-head of needle j is also written as Eq 4 in local coordinate frame Σ_*cj*_ which is set up as the Σ', as shown in Fig 7. However, because the position of needle j varies during the needle-insertion trajectory planning, a local coordinate frame Σ'' attached to the needle j is set up (Fig 7) and it varies with the movement of needle j. The *z*''-axis of Σ'' coincides with the axis of needle j and the origin *o*'' coincides with the tip of the needle j, which is also $P_{Bj}^{'}$. So, the point coordinates [*b*_*x*_, *b*_*y*_, *b*_*z*_]^*T*^ in Σ can be converted by [*b*_cx_, *b*_cy_, *b*_cz_]^T^ in Σ_*cj*_:

$${\left[b_{\text{x}},b_{\text{y}},b_{\text{z}},1\right]}^{\text{T}}=T_{{\text{n}}_{j}}{\left[b_{\text{cx}},b_{\text{cy}},b_{\text{cz}}\right]}^{\text{T}}$$ (8)

where, $T_{{\text{n}}_{j}}$ is the homogeneous transformation matrix from Σ_*cj*_ to Σ:

$$T_{{\text{n}}_{j}}=\left[\begin{array}{cccc} 1 & 0 & 0 & x_{\text{o}j}+\delta_{\text{x}j}\\ 0 & 1 & 0 & y_{\text{o}j}+\delta_{\text{y}j}\\ 0 & 0 & 1 & z_{\text{o}j}+\delta_{\text{z}j}\\ 0 & 0 & 0 & 1 \end{array}\right]\left[\begin{array}{cccc} \text{cos}\;\varphi_{j} & -\text{sin}\;\varphi_{j} & 0 & 0\\ \text{sin}\;\varphi_{j} & \text{cos}\;\varphi_{j} & 0 & 0\\ 0 & 0 & 1 & 0\\ 0 & 0 & 0 & 1 \end{array}\right]\left[\begin{array}{cccc} \text{cos}\;\theta_{j} & 0 & \text{sin}\;\theta_{j} & 0\\ 0 & 1 & 0 & 0\\ -\text{sin}\;\theta_{j} & 0 & \text{cos}\;\theta_{j} & 0\\ 0 & 0 & 0 & 1 \end{array}\right]\left[\begin{array}{cccc} 1 & 0 & 0 & 0\\ 0 & 1 & 0 & 0\\ 0 & 0 & 1 & l/2\\ 0 & 0 & 0 & 1 \end{array}\right]\left[\begin{array}{cccc} \text{cos}(\pi /2) & 0 & \text{sin}(\pi /2) & 0\\ 0 & 1 & 0 & 0\\ -\text{sin}(\pi /2) & 0 & \text{cos}(\pi /2) & 0\\ 0 & 0 & 0 & 1 \end{array}\right]$$ (9)

According to Eqs 8 and 9, the description function $f_{{\text{n}}_{j}}$ of the curved surface of needle j with position *q* in Σ can be obtained.

Once the mathematical descriptions of the obstacles and the needle j are obtained, based on our previous work [34], the collision-free equation ***Φ***' between the needle j and obstacles can be formulated as:

$$\Phi^{'}\equiv \left[\begin{array}{c} \frac{\partial f_{0}(\eta )}{\partial \eta }+\lambda_{1}\frac{\partial f_{{\text{n}}_{j}}(\text{q},\eta )}{\partial \eta }+\lambda_{2}\frac{\partial f_{\text{obs}}(\eta )}{\partial \eta }\\ f_{{\text{n}}_{j}}(\text{q},\eta )+s_{1}\\ f_{\text{obs}}(\eta )+s_{2}\\ \mathrm{LSe}-\mu e\\ {\left\|a_{\text{g}}-b_{\text{g}}\right\|}_{2}^{2}-\gamma^{2}-s_{d}{}^{2} \end{array}\right]=0$$ (10)

where ***a***_*g*_ = [*a*_*x*_, *a*_*y*_, *a*_*z*_]^*T*^ and ***b***_*g*_ = [*b*_*x*_, *b*_*y*_, *b*_*z*_]^*T*^ are the point coordinates on curved surfaces of both the obstacles and needle j in Σ, $\eta ={\left[a_{g}^{T},b_{g}^{T}\right]}^{T}$ is a 6×1 vector, $f_{0}(\eta )={\left\|a_{g}-b_{g}\right\|}_{2}^{2}$; ***λ*** = [*λ*_1_, *λ*_2_]^*T*^ is a 2×1 vector of Lagrange multipliers, ***L*** is a 2×2 diagonal matrix of *λ*_1_ and *λ*_2_, ***S*** is a 2×2 diagonal matrix of the slack variables *s*_1_ and *s*_2_, ***s*** = [*s*_1_, *s*_2_]^*T*^ is a 2×1 vector; ***e*** = [1, 1]^*T*^, and *γ* > 0 is the safe-distance parameter, which can be decided by clinical needs to avoid a needle insertion trajectory too close to important organs. We can set the value based on the clinical experience; *μ* is the barrier parameter and *s*_*d*_ is a slack variable. ***Φ***' is a nonlinear equation set containing 11 equations with 11 variables ***w***_*1*_ = [***η***^*T*^, ***λ***^*T*^, ***s***^*T*^, *s*_*d*_]^*T*^.

Combing Eqs 1 and 10, the extended set of constraint equations $\tilde{\Phi }$ with 13 equations is obtained:

$$\tilde{\Phi }\equiv \left[\begin{array}{c} \Phi \\ \Phi ' \end{array}\right]=0$$ (11)

The analytical criterion of needle CFRW for needle j can be given by [34]:

$$\tilde{G}(\tilde{x})\equiv \left[\begin{array}{c} \tilde{\Phi }(u,\tilde{z})\\ {\tilde{\Phi }}_{\tilde{z}}^{T}(u,\tilde{z})\tilde{\xi }\\ {\tilde{\xi }}^{T}\tilde{\xi }-1 \end{array}\right]=0$$ (12)

where ***u*** = [*x*, *y*]^T^, $\tilde{z}={\left[a_{x},a_{y},a_{z},b_{x},b_{y},b_{z},\lambda_{1},\lambda_{2},s_{1},s_{2},s_{d},v_{1},v_{2}\right]}^{T}$, $\tilde{\zeta }={\left[\zeta_{1},\cdots \zeta_{13}\right]}^{T}$ is a unit 13-dimensional vector, because $\tilde{x}={\left[u^{T},{\tilde{z}}^{T},{\tilde{\xi }}^{T}\right]}^{T}\in R^{28}$ and Eq 12 contains 27 equations, $\tilde{G}(\tilde{x}):R^{28}\to R^{27}$ is a 1D curve on *z*_*h*_-plane.

Thus, the boundary of the CFRW of needle j is obtained as a 1D curve in the *z*_*h*_-plane. The boundaries of CFRW of needle j in other *xy*-planes can be obtained in the same way with different values of h. The 3D boundary of CFRW of the needle j in space comprises a group of 1D boundaries on *xy*-planes. If there are several obstacles, the needle CFRW is the intersection of the needle CFRWs for each obstacle.

Once the needle CFRW is solved, the optimal needle insertion trajectory can be obtained based on the optimization criterion in the needle CFRW.

#### Optimal needle-insertion trajectory planning

From the obtained boundary of the needle CFRW, the optimal needle-insertion trajectory can be selected according to designed optimization criteria which are critical for the planning. The mission of the RALTCT system is highest probability to hit the target and minimum invasiveness to the patient. Taking into account the deformation of soft tissue and other random errors, a long needle-insertion trajectory suffers from potentially missing the target. To this end, the shortest distance between the entry point and the target point is proposed as an optimization criterion of the needle-insertion trajectory in this paper. That is, the optimal needle-insertion trajectory planning is to find the optimal entry point, which determines the shortest distance between the entry point and the target within the obtained needle CFRW. This will ensure that no important organs are approached within the clinical safe distances of the needle-insertion trajectory and also some clinical constraints—such as insertion depth, insertion angle, and the distance to important organs—are set by parameters in the analytical criteria for the needle CFRW.

Furthermore, mathematically, the solution of the optimal entry point is equivalent to a point search on the bounded curved surface (the patient’s abdominal epidermis), under the constraint of the local minimum distance between the searched point and a given point (the target). Essentially, this is a local optimal problem. To solve this problem, a discrete grid method is used in this paper because of its insensitivity to initial conditions and low computational complexity.

The optimal needle insertion trajectory is the shortest one; that is, the shortest distance between the entry point *P*_*Aj*_ and the target point $P_{Bj}^{'}$. Additionally, the distance between the needle insertion trajectory and the important organs should be further than the clinical safe distance to avoid potentially fatal injury. Thus, the candidate entry point on the curved surface (the patient’s abdominal epidermis) has to be found within the boundary of the needle CFRW under the constraint of the shortest distance to point $P_{Bj}^{'}$. Consequently, the multiple-needle insertion-trajectory planning can be implemented automatically.

Here, we assume that ***S***(*u*, *v*) = [*x*(*u*, *v*), *y*(*u*, *v*), *z*(*u*, *v*)]^T^ is the parametric equation of the bounded curved surface of the abdominal epidermis, where *u* ∈ [*u*_min_, *u*_max_], *v* ∈ [*v*_min_, *v*_max_]. The range of *u*, *v* can be determined by the needle rotation angle *θ*. The mathematical model of the solution of the optimal entry point ***P***' can be written as:

$$d_{\text{min}}(u,v)=\text{min}\left\{\left\|P_{Bj}^{'}P_{n}^{'}\right\|\right\}=\text{min}\left\{\left\|r_{\left(u,v\right)}-r_{P_{Bj}^{'}}\right\|\right\}$$ (13)

where *r*_(*u*, *v*)_ is the radius vector of the point $P_{n}^{'}$ on the curved surface, and $r_{P_{Bj}^{'}}$ is the radius vector of the target point $P_{Bj}^{'}$.

**Fig 8:**
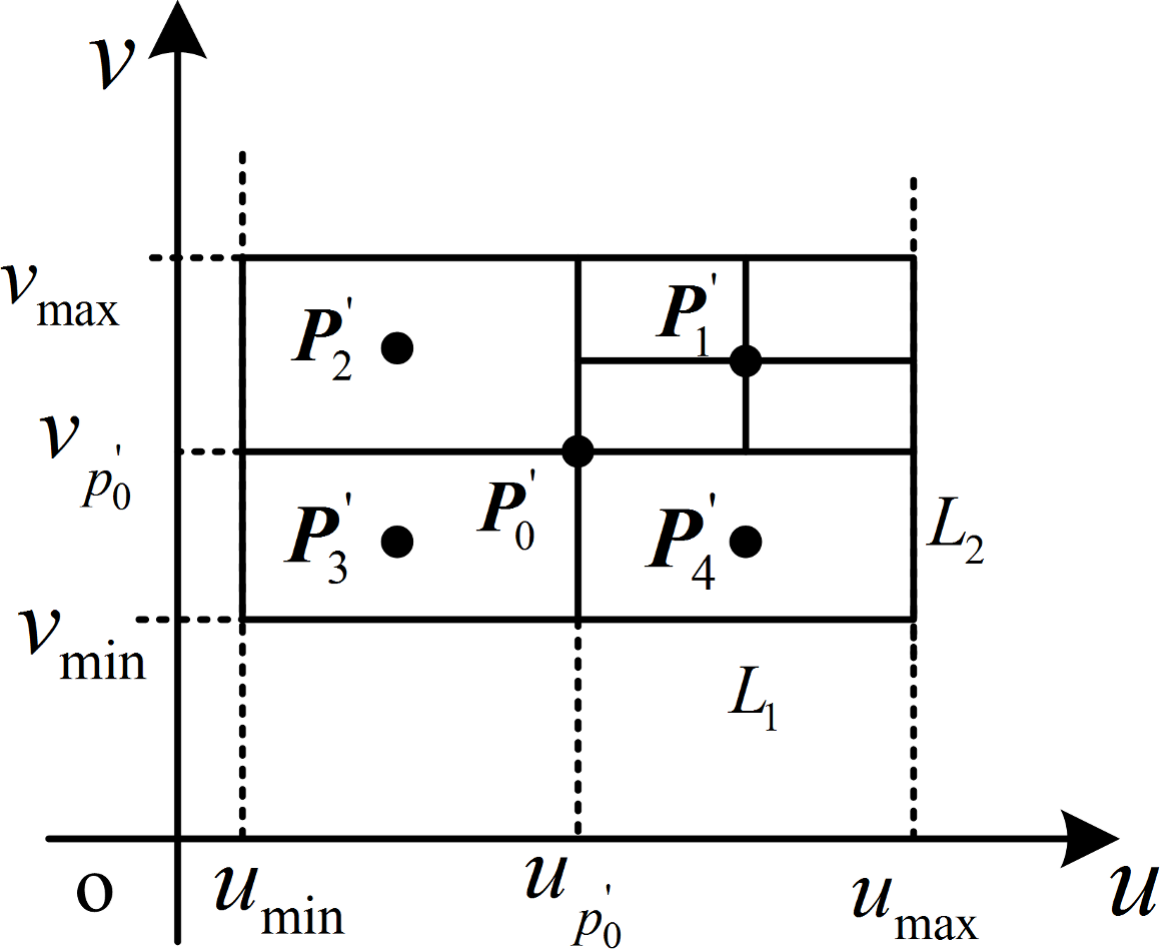
Schematic diagram of the grid method’s solution.

**Fig 9:**
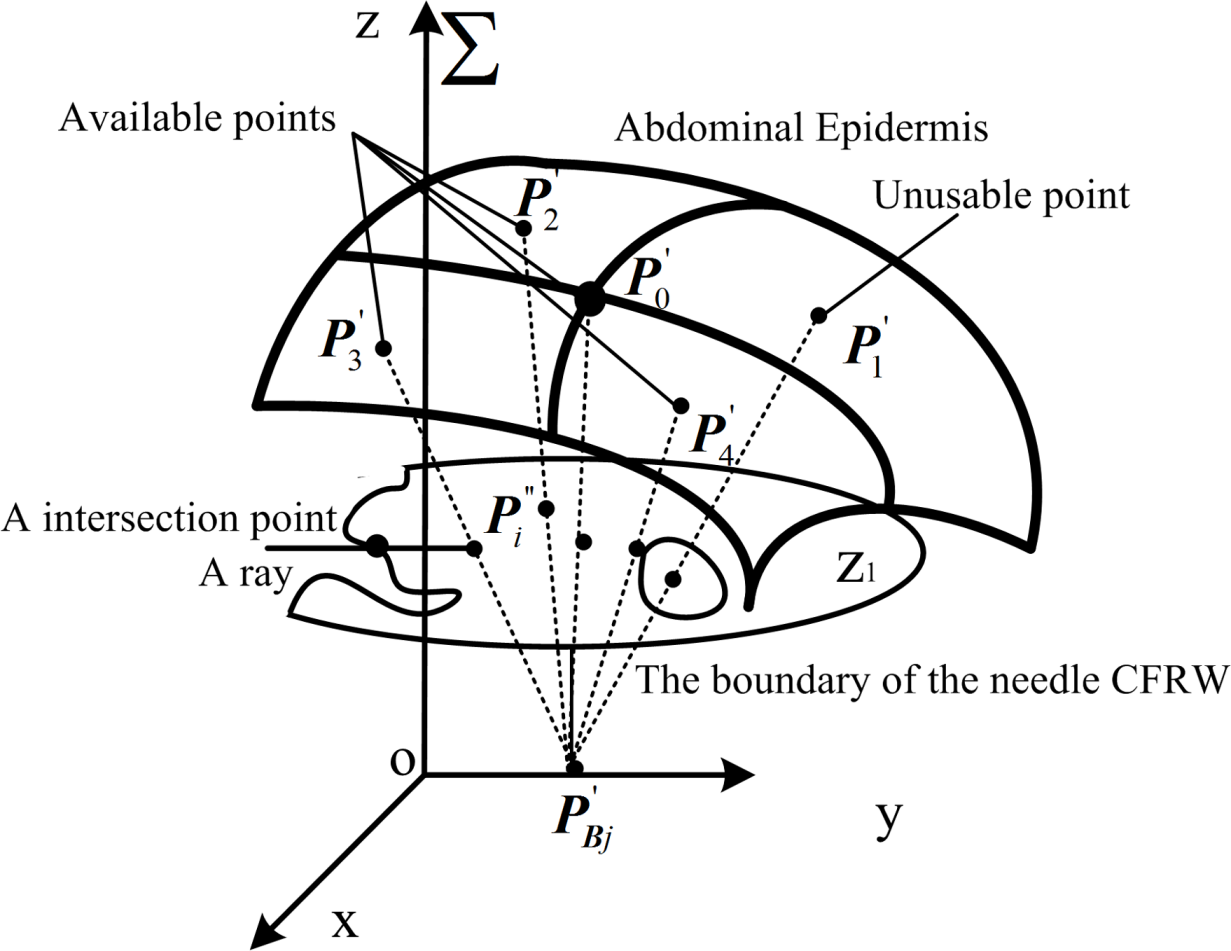
Schematic diagram of the judgment as to whether the needle-insertion trajectory is within the boundary of the needle CFRW.

The procedure of the optimal needle-insertion trajectory planning is realized as follows:

1. select the initial point $P_{0}^{'}$ corresponding to $u_{P_{0}^{'}}=(u_{\text{min}}+u_{\text{max}})/2,v_{P_{0}^{'}}=(v_{\text{min}}+v_{\text{max}})/2$. The original iterative intervals in the direction *u*, *v* are *L*_1_ = (*u*_max_ − *u*_min_) / 2, *L*_2_ = (*v*_max_ − *v*_min_) / 2, respectively;
2. obtain four grid regions with the initial point in the center, as shown in Fig 8. The centers of each grid region are $P_{i}^{'}(i=1,2,3,4)$ with parameter coordinates $\left(u_{P_{0}^{'}}+0.5L_{1},v_{P_{0}^{'}}+0.5L_{2}\right)$, $\left(u_{P_{0}^{'}}-0.5L_{1},v_{P_{0}^{'}}+0.5L_{2}\right)$, $\left(u_{P_{0}^{'}}-0.5L_{1},v_{P_{0}^{'}}-0.5L_{2}\right)$, and $\left(u_{P_{0}^{'}}+0.5L_{1},v_{P_{0}^{'}}-0.5L_{2}\right)$, respectively.
3. decide whether line segment $P_{i}^{'}P_{Bj}^{'}$ (*i* = 0,1,2,3,4) (needle-insertion trajectory) is within the 3D boundary of the needle CFRW. Without loss of generality, we can simplify this problem by deciding whether the intersection points $P_{i}^{''}$ between line segment $P_{i}^{'}P_{Bj}^{'}$ and *z*_1_-plane (it is also achieved on other z-plane with the corresponding *h* ∈ [0, *h*_max_]) are within the corresponding boundary of the needle CFRW or not. The boundary of the needle CFRW is a closed 1D manifold on the *z*_1_-plane; thus, the ray method [35–36] is used because of its ability to handle holes within the boundary. The ray method determines whether the point is within the boundary from the number of the intersection points between the boundary and a ray through the point in any direction (Fig 9). Because of the analytical expression of the boundary of the needle CFRW Eq 12, the judgment can be implemented conveniently. If intersection point $P_{i}^{''}$ is within the boundary, then corresponding point $P_{i}^{'}$ is available; otherwise, corresponding point $P_{i}^{'}$ is unusable.
4. calculate the distances between point $P_{\text{B}j}^{'}$ and point $P_{i}^{''}$. Among these distances, there must be a minimum distance *d*_min_ ($d_{\text{min}}=\text{min}\left\{\left\|P_{\text{B}j}^{'}P_{i}^{''}\right\|\right\}$) and a corresponding point $P_{\text{A}j}^{'}$ on the surface.
5. decide whether the termination condition is satisfied:
  $$\frac{\left\|P_{\text{A}j}^{'}P_{\text{B}j}^{'}\times n_{P_{\text{A}j}^{'}}\right\|}{\left\|P_{\text{A}j}^{'}P_{\text{B}j}^{'}\|\|n_{P_{\text{A}j}^{'}}\right\|}\leq \delta \;\text{or}\;L\leq \varepsilon$$ (14)
  where $n_{P_{\text{A}j}^{'}}$ is the normal vector of $P_{\text{A}j}^{'}$, *δ* is the convergence precision, *ε* is the step precision, *L* = max(*L*_1_, *L*_2_), if the termination condition is satisfied, the next step would be executed; if not, step (2) would be executed and select $P_{\text{A}j}^{'}$ as a new initial point $P_{0}^{'}$. The intervals *L*_1_ and *L*_2_ will be reduced by half as new intervals for further mesh refinement.
6. The closest distance between target point $P_{\text{B}j}^{'}$ and surface ***S*** is *d*_min_ and the corresponding closest point on ***S*** is $P_{\text{A}j}^{'}$.

Finally, the optimal needle-insertion trajectory is obtained.

## Results and Discussion

A simulation experiment and an *in vitro* experiment integrating all the procedures were carried out to evaluate the proposed automatic surgical planning method.

### Simulation results of multiple-needle insertion trajectory planning

**Fig 10:**
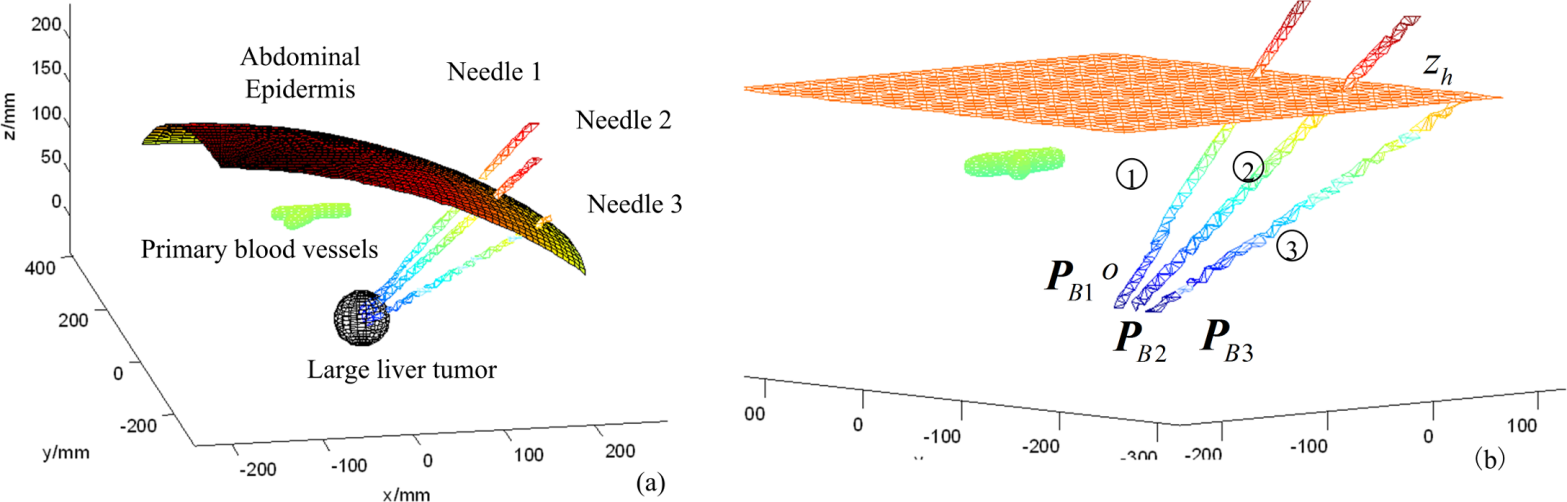
Schematic diagram of multiple-needle surgical planning environment. (a) The abdominal operation environment. (b) The information of target points and insertion order.

Without loss of generality, a three-needle insertion trajectory planning was simulated using the primary blood vessels as the obstacles. When the number of inserted needles is increased, we should only repeat the procedures above to solve the needle CFRW and the corresponding optimal needle-insertion trajectory within the needle CFRW. The abdominal operation environment is shown in Fig 10(A), where ***P***_*B*1_ = [0, 0, 0]^*T*^ mm, ***P***_*B*2_ = [0, −15, 0]^*T*^ mm, and ***P***_*B*3_ = [0, −30, 0]^*T*^ mm are the three target points for needle 1, needle 2, and needle 3, respectively. The order of needle insertion is needle 1, needle 2, and needle 3, in turn. We described the needle and blood vessels by the super-quadric equation, and described the abdominal epidermis by a parametric equation. The needle-insertion trajectory planning task is to seek three optimal entry points *P*_*A*1_, *P*_*A*2_, and *P*_*A*3_, corresponding to the optimal needle insertion trajectories, based on the needle CFRW. The optimal needle-insertion trajectory will not interfere with the obstacles, especially the previously inserted needles.

**Fig 11:**
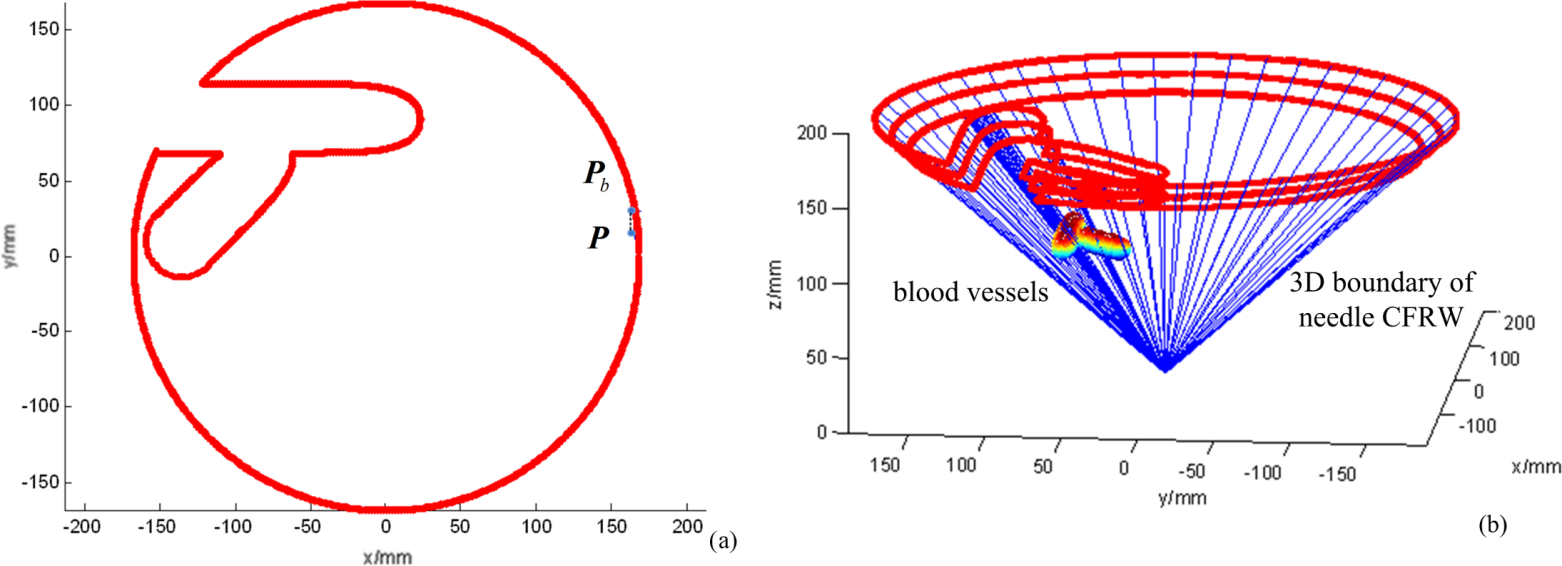
The boundaries of needle 1’s CFRW. (a) The 2D CFRW for needle 1 with z = 150. (b) The 3D CFRW for needle 1 with z = 150, 160, 170 mm.

Based on the method above, the 1D boundary of the needle CFRW in the *z*_*h*_-plane can be described with Eq 12. Then, the continuation method [37] was used to calculate Eq 12, benefitting from its insensitivity to the initial value for the parameterized non-linear equation. The program was compiled in Visual C++6.0. On the *xy*-plane with *z* = 150 mm, we chose initial search point ***P*** = [164.9218, 15.4162]^*T*^ within the workspace to solve Eq 12. ***P***_*b*_ = [164.9218, 30.2989]^*T*^ mm is the point on the boundary obtained by searching from ***P*** along the [0, 1]^*T*^ direction. Setting *γ* = 1.5 mm and *μ* = 0.1, the simulation result is shown in Fig 11. Fig 11(A) shows the needle CFRW for needle1 in the *xy*-plane with z = 150. Fig 11(B) shows the boundaries of CFRW for needle1 in the *xy*-planes with *z* = 150,160,170 mm.

**Fig 12:**
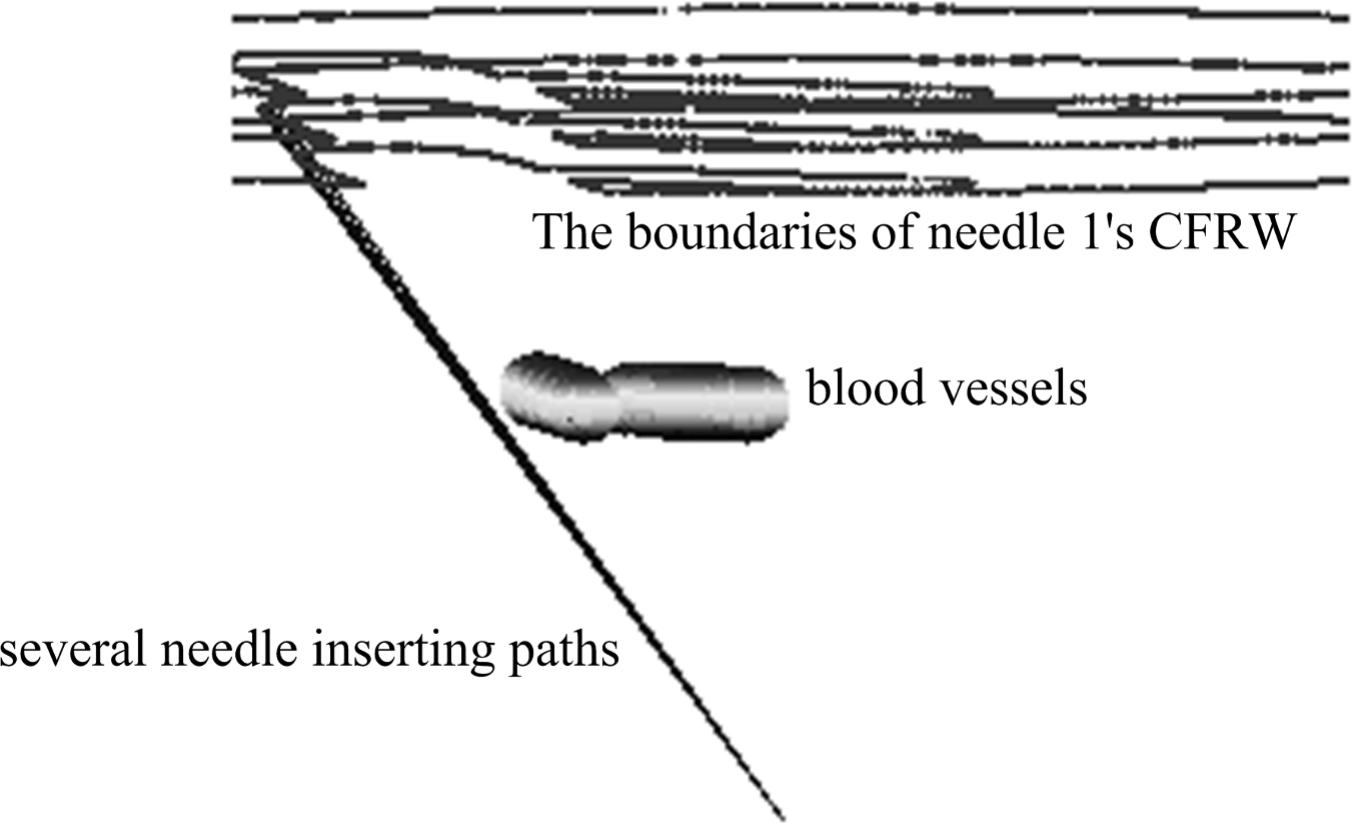
The local enlarged map.

As shown in Fig 12, the lines are the needle-insertion trajectories, the entry points of which are on the boundaries. We calculated the closest distances between the lines and blood vessels, all of which are 1.5 mm. These results are consistent with the settings above, such as *γ* = 1.5. This also confirmed that the safe distance *γ* could be adjusted according to the actual operation to control the closest distance between the needle-insertion trajectory and obstacles. Also, we can see that the needle-insertion trajectories in the needle CFRW will not interfere with obstacles.

**Fig 13:**
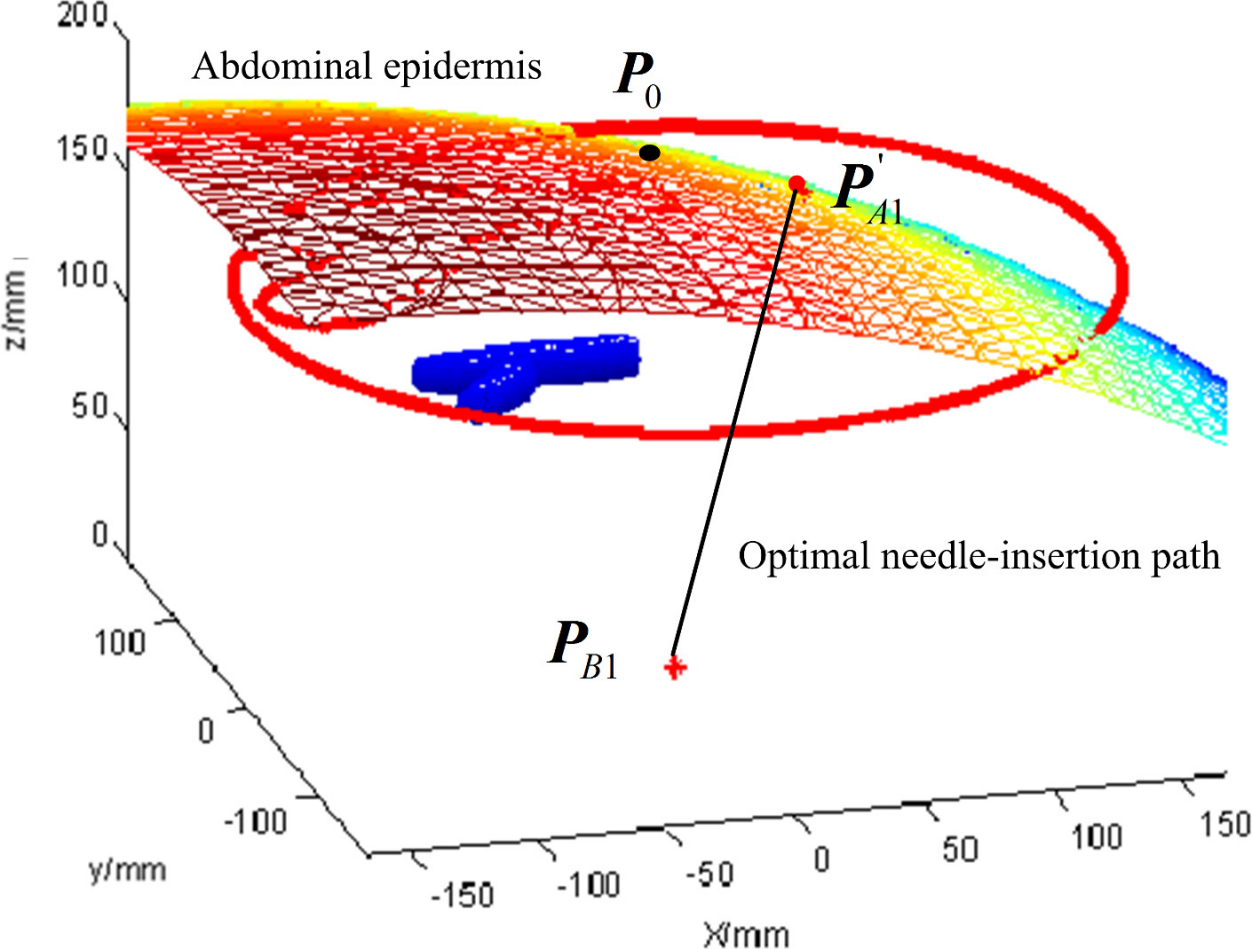
Optimal needle-insertion trajectory planning.

Based on the obtained boundaries of the needle 1 CFRW, the optimal needle-insertion trajectory is planned by the grid method with the optimization criteria. The optimization algorithm was implemented on MATLAB. Setting the convergence precision *δ* = 1×10^−5^ and the step precision *ε* = 1×10^−4^, the planned result is shown in Fig 13. *P*_0_ is the initial point and $P_{A1}^{'}$ is the planned optimal entry point. Because $P_{A1}^{'}$ is within the boundary of the needle CFRW, the planned needle-insertion trajectory formed by the line segment connecting $P_{A1}^{'}$ and target ***P***_*B*1_ would not damage the primary blood vessels and satisfy the clinical constrains.

**Fig 14:**
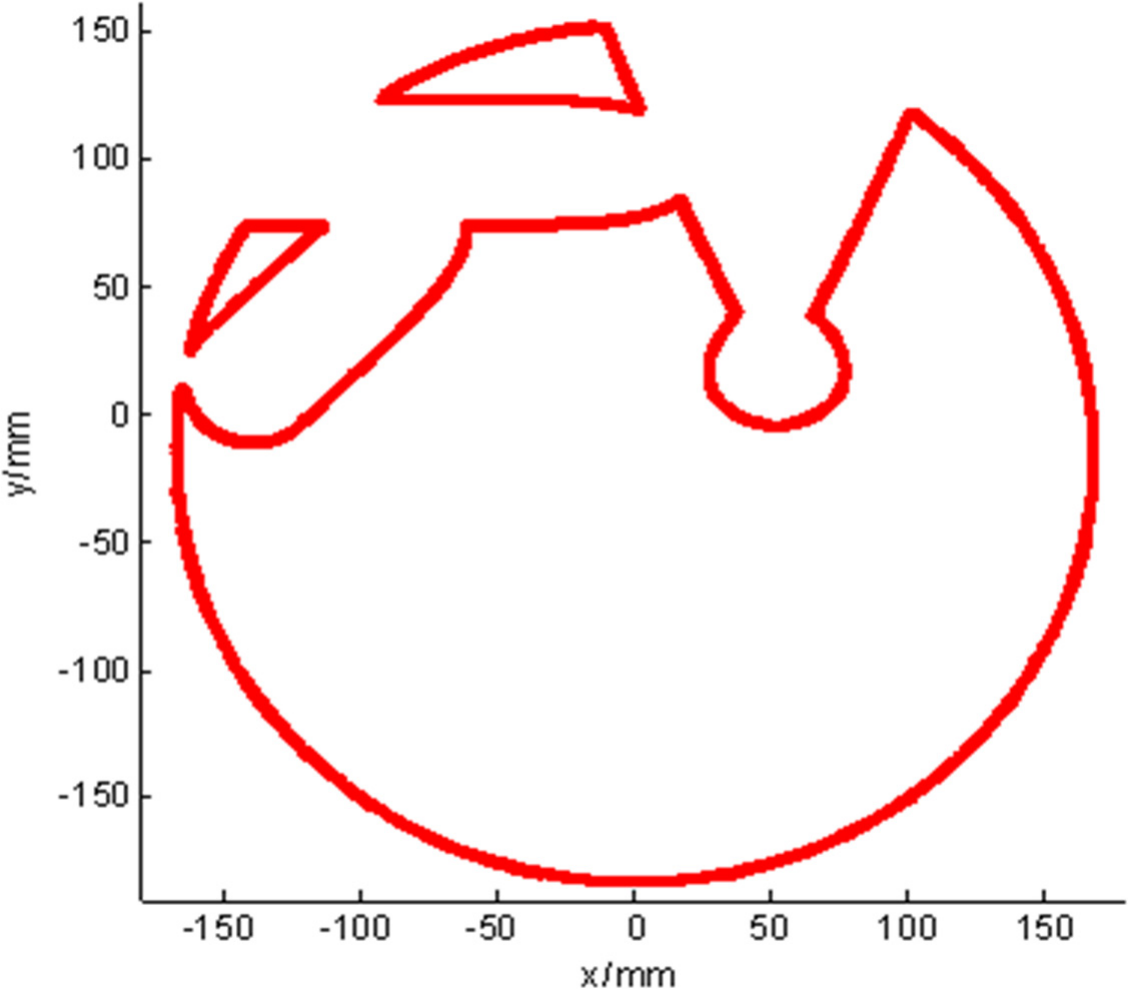
The needle2 CFRW.

**Fig 15:**
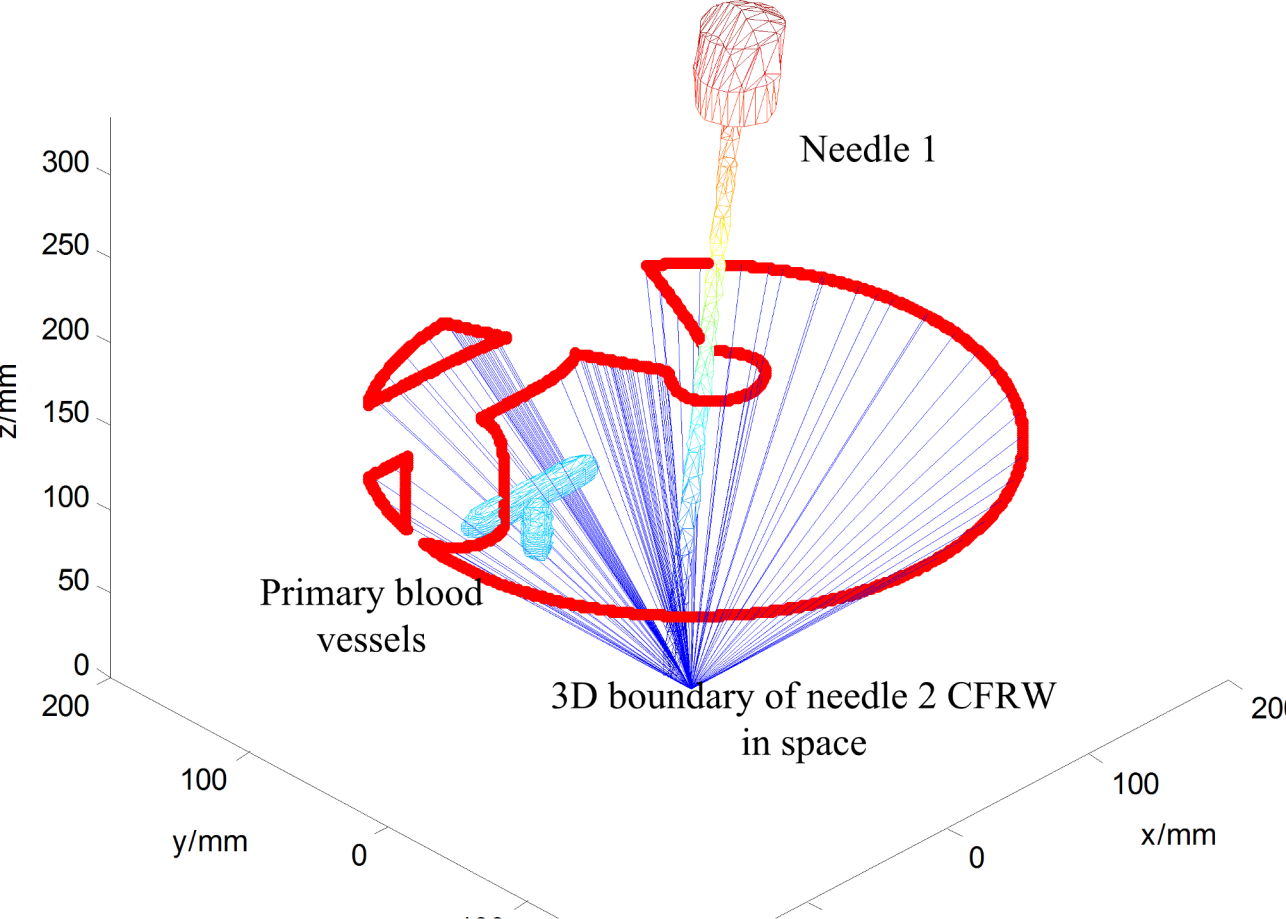
The 3D boundary of CFRW for needle 2.

**Fig 16:**
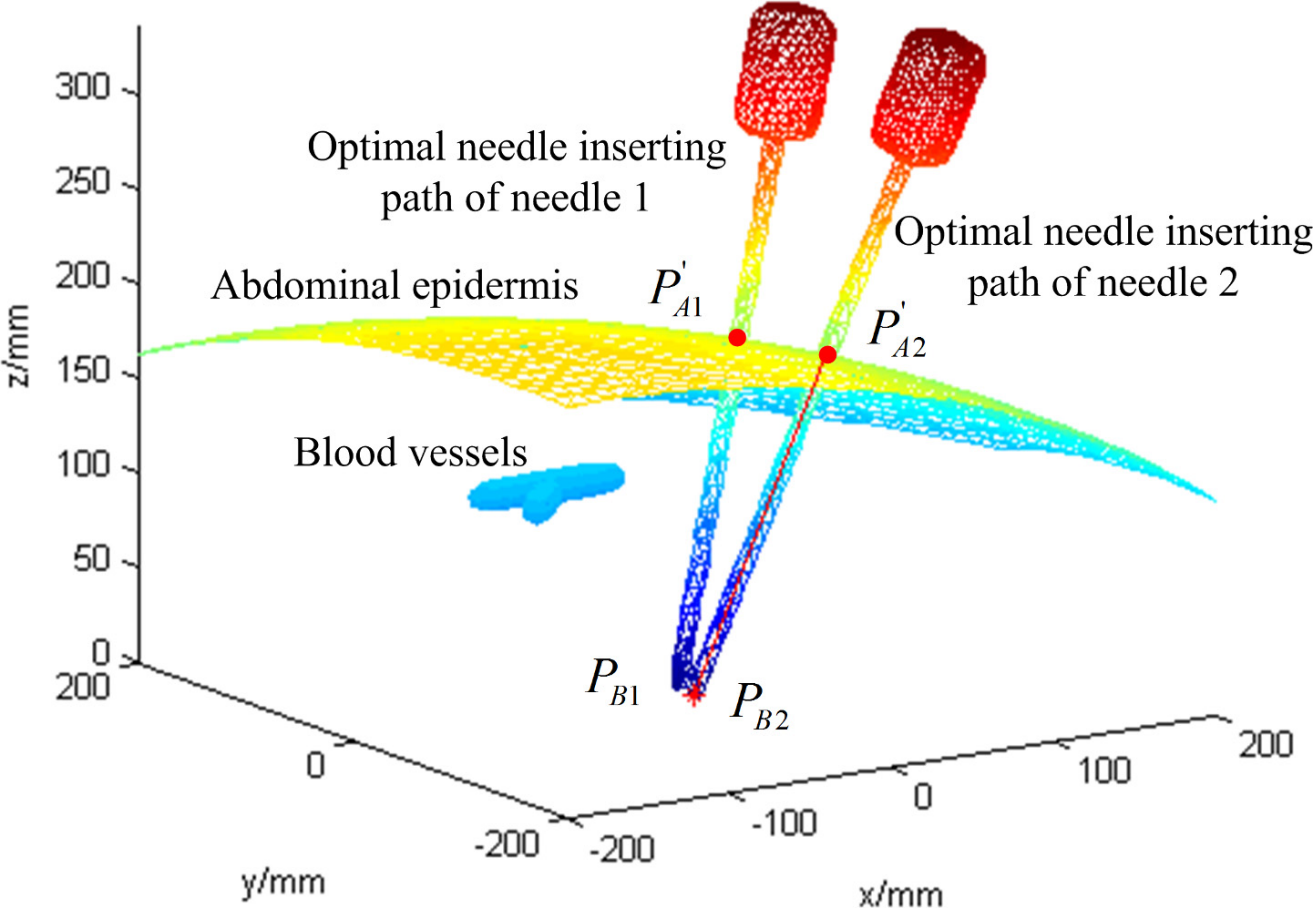
Optimal needle-insertion trajectories for needle 1 and needle 2.

After the optimal needle-insertion trajectory planning for needle 1, the position of the inserted needle 1 would be calculated. Then, the inserted needle 1 would be a new obstacle during insertion of needle 2. For the calculation of the needle 2 CFRW, first, we solve the needle 2 CFRWs for blood vessels as the obstacle and then the inserted needle 1 as an obstacle. Then, the needle 2 CFRW is the intersection of the two obtained CFRWs. The result is shown in Fig 14 and the corresponding 3D result is given in Fig 15. Sequentially, the optimal needle insertion trajectory of needle 2 is selected in the same way (Fig 16). The simulation results indicate that the two planned optimal needle-insertion trajectories will not damage blood vessels and the two inserted needles will not interfere with each other.

**Fig 17:**
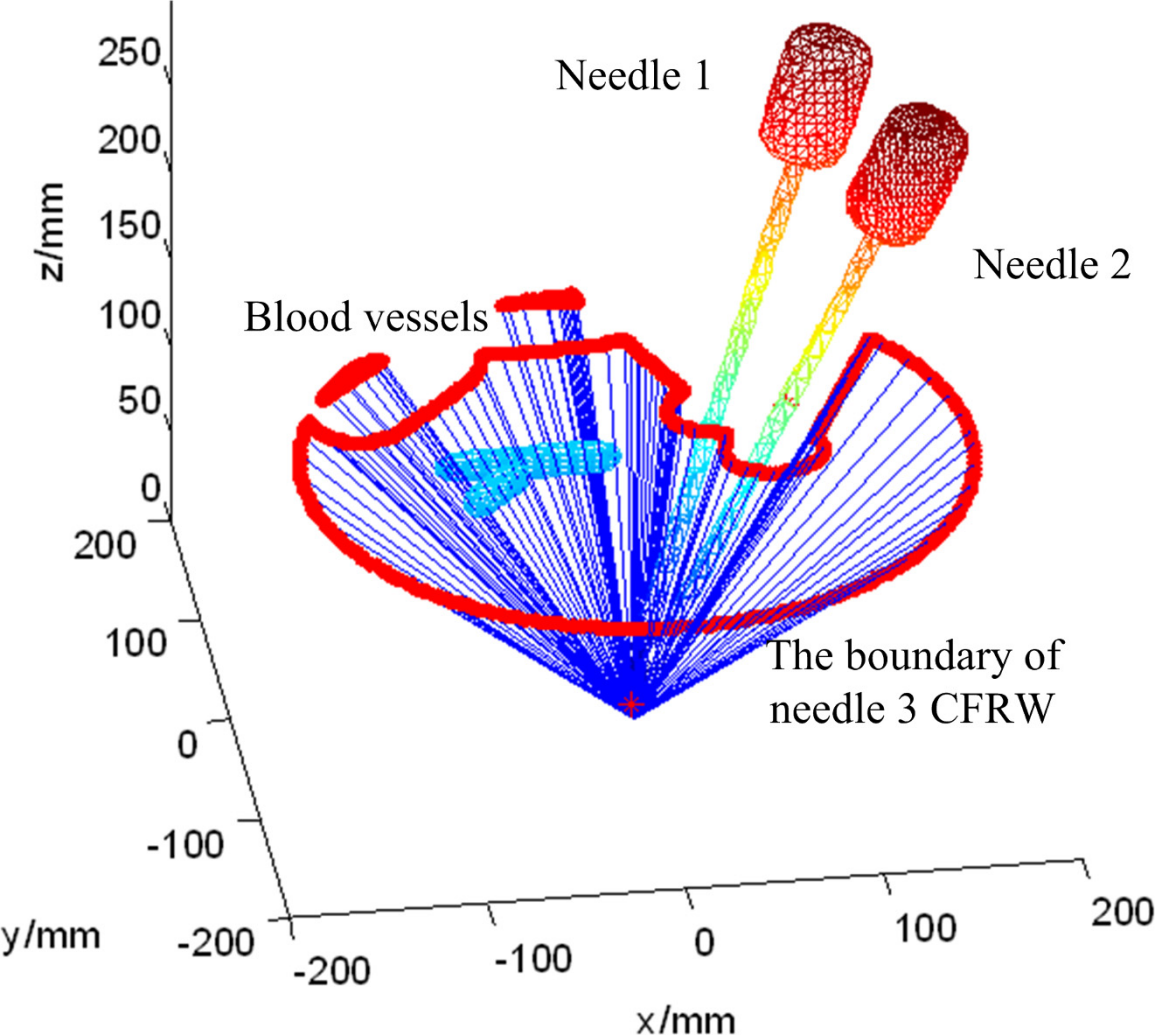
The 3D boundary of needle 3’s CFRW.

**Fig 18:**
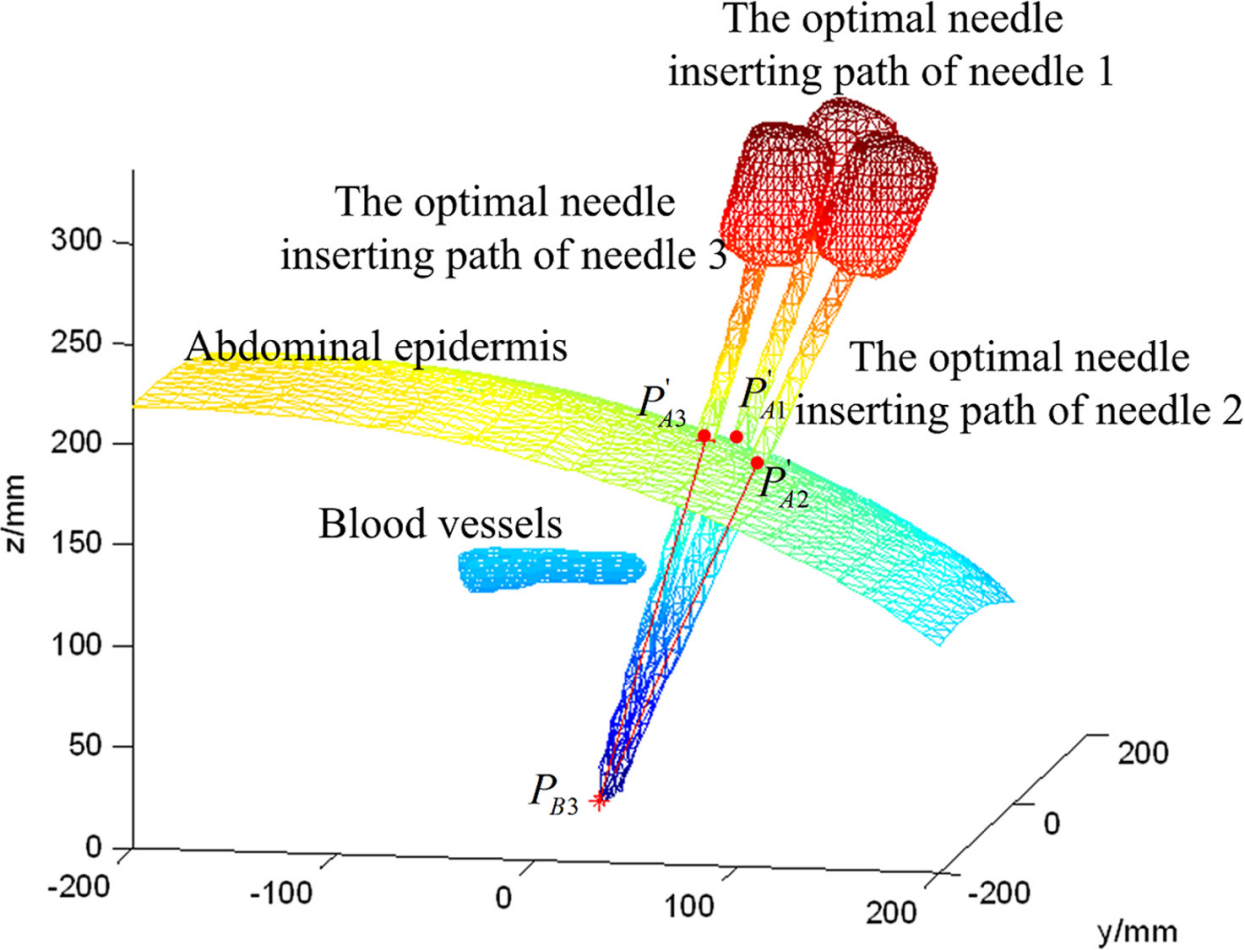
The optimal needle-insertion trajectories of the three needles.

For the needle-insertion trajectory planning for needle 3, the blood vessels, inserted needle 1 and needle 2 are all obstacles and then the above steps are repeated to solve the needle 3 CFRW and the optimal needle-insertion trajectory successively. The results are shown in Figs 17 and 18. $P_{A3}^{'}$ is the entry point corresponding to the optimal needle-insertion trajectory. The simulation results indicate that all of the three optimal needle-insertion trajectories are within the corresponding needle CFRW and they do not interfere with each other or damage the blood vessels.

Thus, the three optimal needle-insertion trajectories of the three needles have been calculated. The simulation of multiple-needle surgical planning for three-needle surgery has been demonstrated.

The simulation results indicate that the proposed method is applicable to different operation environments, as long as the obstacles can be represented by implicit functions. Thus, the deformation of the liver caused by needle insertion can be considered during planning. Based on the method in paper [38], the point cloud of obstacles and a new target can be obtained. According to the point cloud, the implicit function describing the obstacles can be calculated by a fitting algorithm [39]. The proposed method is also independent of obstacle size, position, and the number of required needles. For an increased number of required needles, the planning procedure is repeated in the same way.

### *In vitro* experiment results of multiple-needle-insertion trajectory planning for a porcine liver

We also tested the feasibility and effectiveness of the proposed multiple-needle surgical planning by using an *in vitro* experiment. One objective of the *in vitro* experiment is to test whether the needle insertion trajectories in the needle CFRW interfere with the obstacles. Another objective is to evaluate the compensation for the target moving, which is intended to reduce the impact of target motion (mainly introduced by deformation of liver during insertion and respiratory motion in clinical surgery) on the accuracy of needle placement.

**Fig 19:**
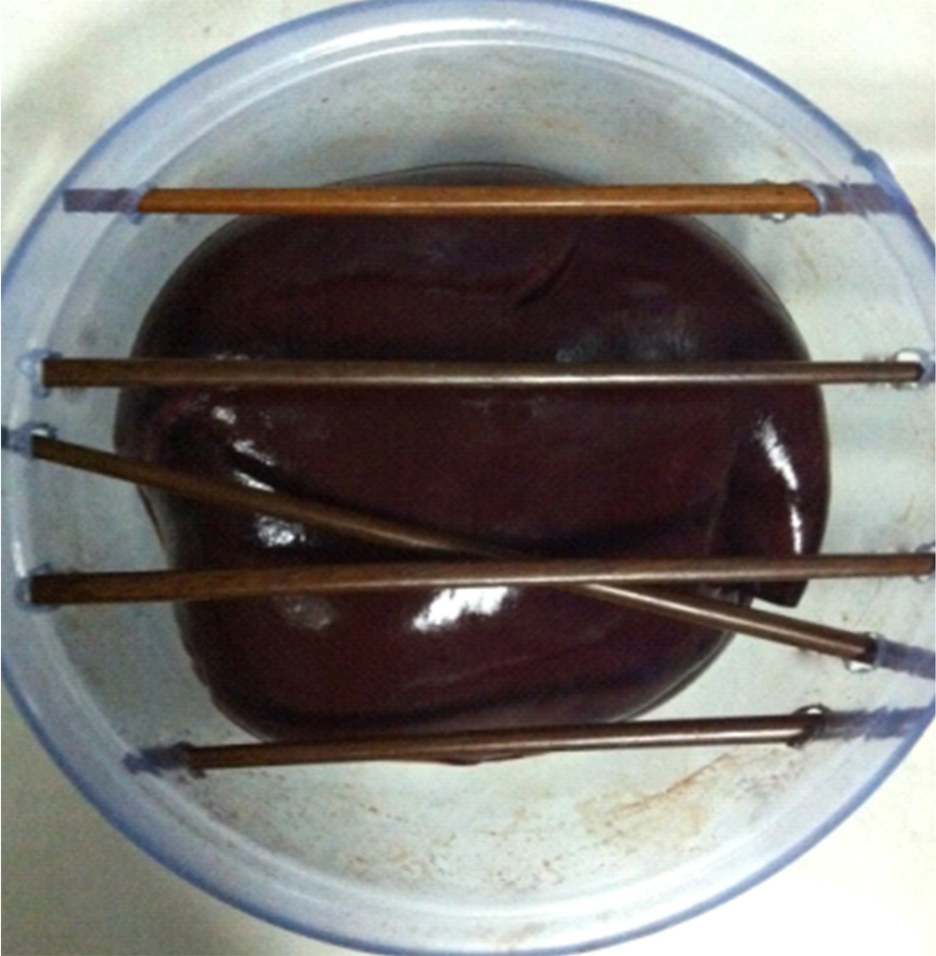
The surgery environment of the *in vitro* porcine liver.

The surgery environment of the *in vitro* experiment for a porcine liver is shown in Fig 19. A 32-cm-diameter plastic box, 3mm thick, was used to contain fresh pieces of *in vitro* porcine liver of two 55-Kg swine, about 1.4 kilogram, which was gotten from the SPF level animal laboratory of the animal laboratory center in Navy general hospital of PLA. Then, a 3-cm-diameter artificial tumor was embedded in porcine liver, which was fabricated by filling molten agar into a spherical injection mold as described in [4]. Several obstacles were present on the liver to mimic the ribs and blood vessels in the abdominal cavity. Based on the size of the agar sphere and multiple-needle surgical planning, two needles were to be placed with the RALTCT system. In this experiment, the main functions of the multiple-needle surgical planning will be verified. For example, the correctness of collision-free needle-insertion trajectories, planning for multiple needles, and the displacement compensator of the target caused by the liver deformation will be verified.

The procedure for this experiment follows the general workflow of a robot-assisted multiple-needle surgery and is outlined as follows:

1. *Data acquisition*. Manually scan the phantom with the US probe, while recording the 2D US images and the corresponding EM tracker data. The positions of dispersed points on obstacles are also obtained by the EM tracker.
2. *Segmentation and modeling*. Manually select the tumor boundary in one or several 2D US images. Other images can be segmented automatically by the improved Lever-Set method [4]. Then, the 3D model of the tumor can be reconstructed using the surgical navigation software. The number of needles and the corresponding positions of targets were defined by user. The mathematical models of obstacles are described by implicit functions based on the disperse points obtained in the last step.
3. *Surgical planning*. Calculate the first needle CFRW. Select the corresponding optimal needle-insertion trajectory in the needle CFRW and the corresponding entry point. Analyze the deformation of the liver [38]. Solve the displacement of the targets and update the corresponding information. According to the compensated position of the target, calculate the second needle CFRW and solve the corresponding optimal needle-insertion trajectory. Also, the entry point for the second needle is obtained in this step.
4. *Registration*. Determine the transformation matrix between the preoperative model and the intraoperative model with the ICP algorithm [4] based on the obstacle information. Then, transform the entry points corresponding to the planned optimal needle-insertion trajectories and the targets to the coordinate system of the robot.
5. *Robot motion*. As shown in Fig 1, a 5-DOF needle-driven robot with a needle-guiding device as its end-effector is used to place the needle. According to the target and the entry point corresponding to the planned optimal needle-insertion trajectory, the end effector will be moved to the desired position by the robot through inverse kinematics. Then, the needle can be inserted to the target location manually. Thereafter, the inserted needle can be loosened from the end-effecter. Then, the robot is switched to passive mode and moved away from the inserted needle. (The robot has two modes: active and passive. The two modes can be switched freely. Generally, the active mode will be selected for needle placement. Once emergence occurs or the operation is finished, the passive mode will be selected and the robot can be moved away from the patient by the surgeon [4].) Repeat the operation to insert the following needles.
6. *Assessment*. Remove the agar sphere and assess the needle insertion accuracy.

During the insertion, we designed some measures to assess the effectiveness of the compensation for the target, based on the analysis of liver deformation in the experiment. The parameters of the first needle insertion, such as insertion depth, were decided manually by the experimenter. At the same time, the insertion was monitored by 2D US imaging. The insertion depth of the second needle was decided by the surgical planning, which was compensated based on the analysis of liver deformation, while the insertion was monitored by means of a depth marker on the needle.

**Fig 20:**
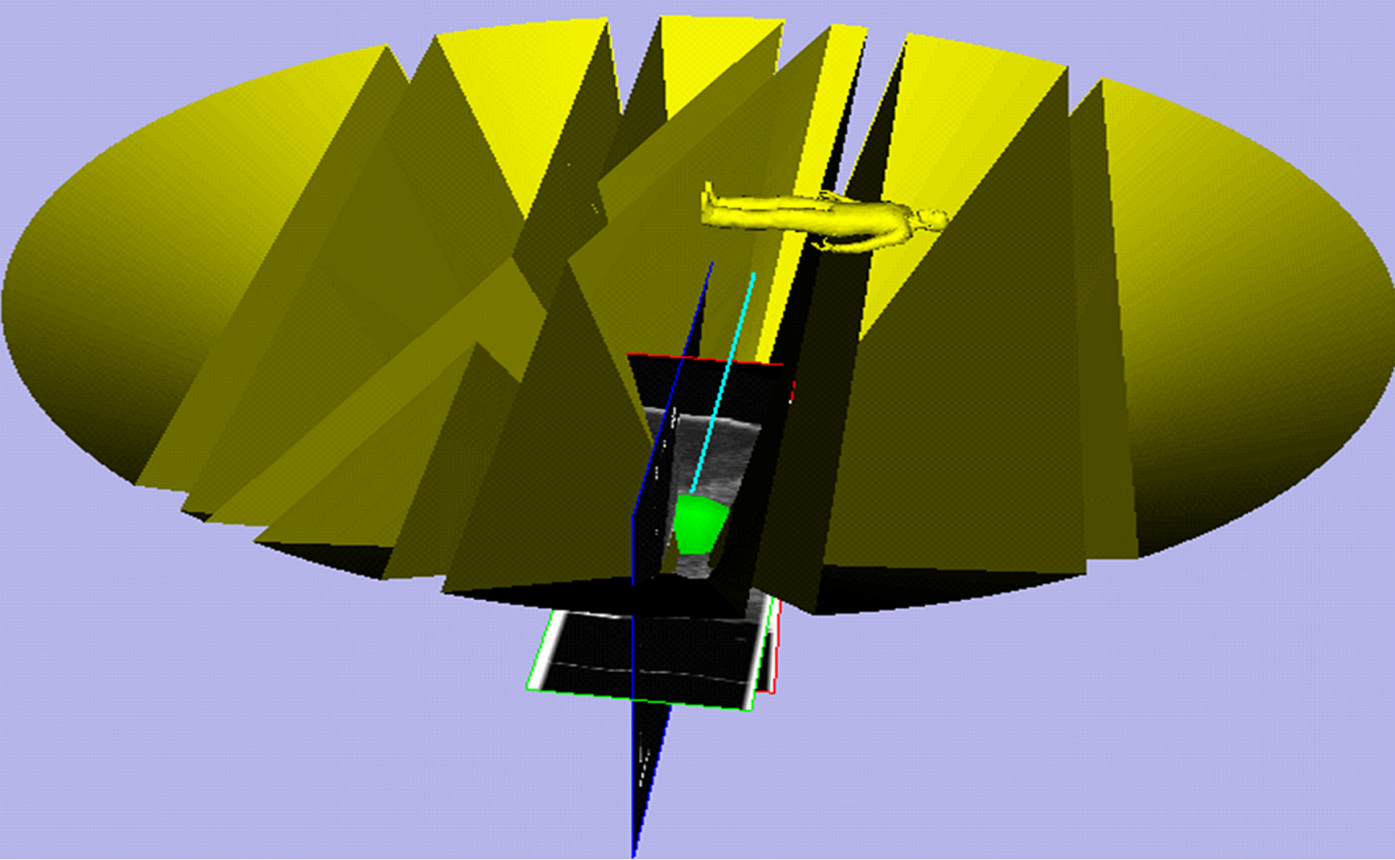
Three-dimensional scenes from the surgical navigation software for the first needle surgical planning.

**Fig 21:**
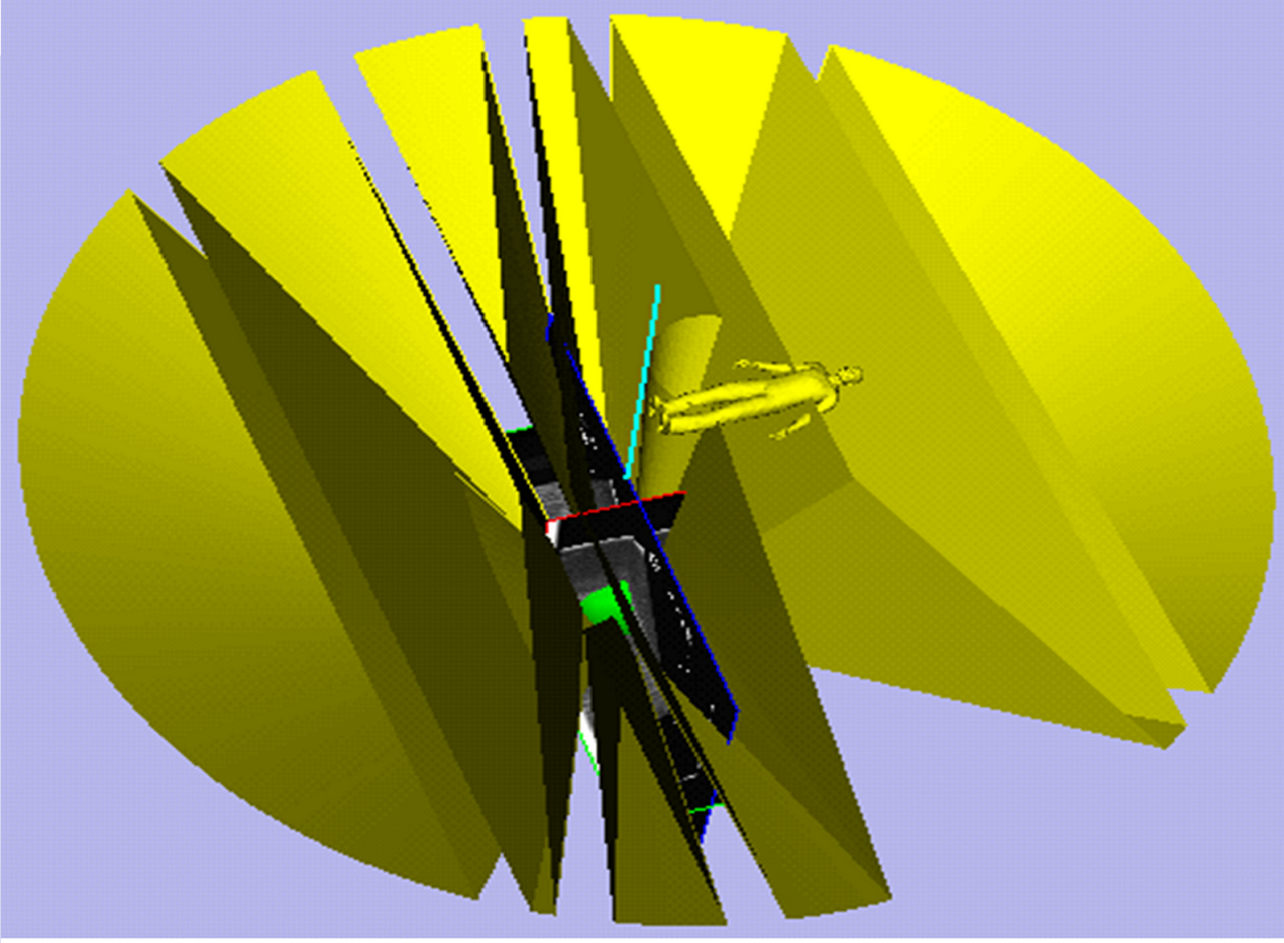
Three-dimensional scenes from the surgical navigation software for the second needle surgical planning.

Thanks to the EM tracking of the needles, the real-time needle insertion trajectory and the planned trajectory can be visualization at the same time in the surgical navigation software. The 3D scenes of the surgical navigation software for the two needle surgical planning steps are shown in Figs 20 and 21. The needle CFRW is the feasible region for the needle-insertion trajectory. The optimal needle-insertion trajectory is planned automatically within this region. The entry point corresponding to the target can also be selected manually in this region easily, when certain conditions are present during surgery.

**Fig 22:**
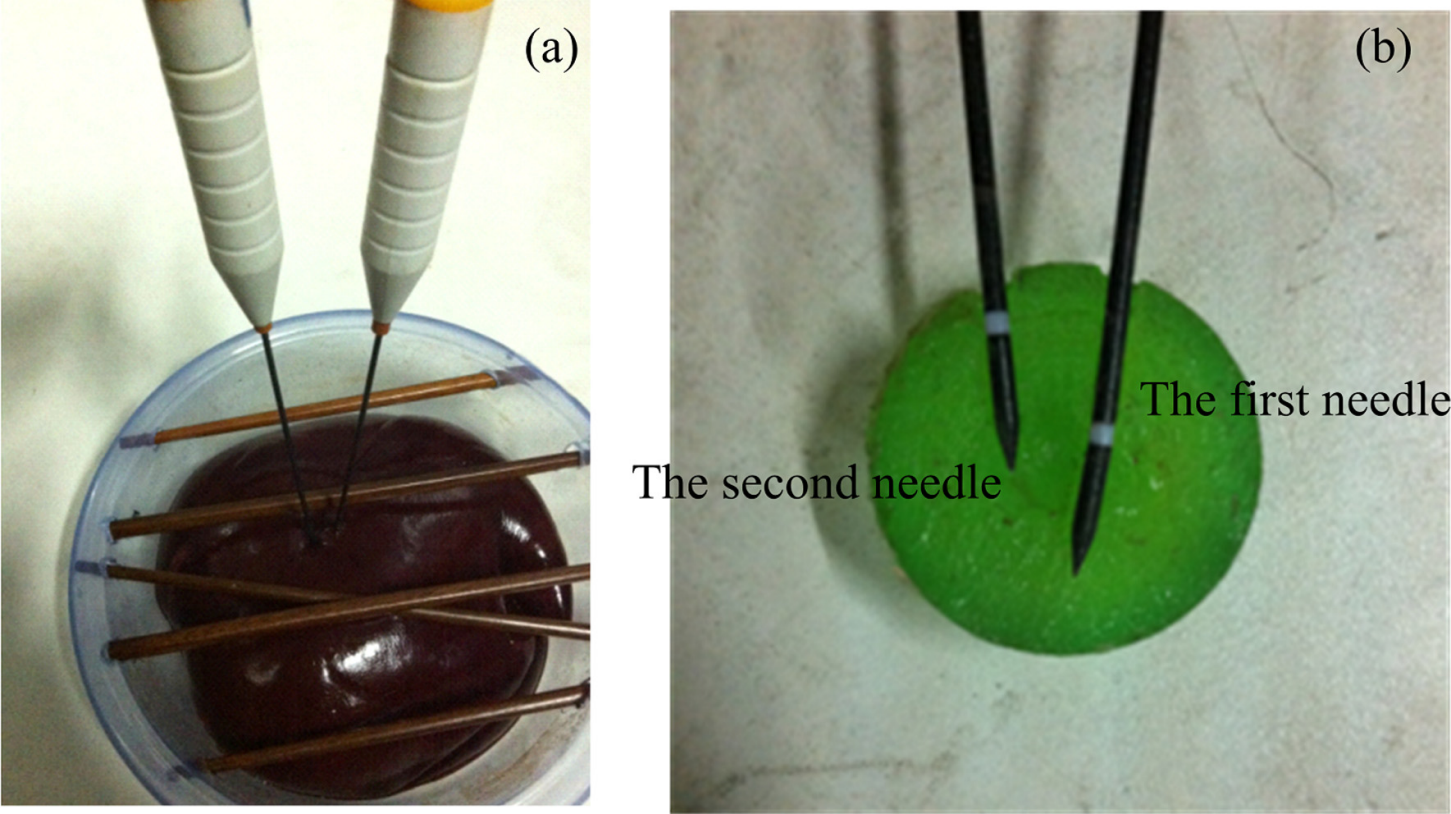
Results of the 3-cm-diameter artificial tumor experiment. (a) The outside appearance of experiment. (b) the insertion accuracy of the two needle.

Results of the experiment are shown in Fig 22. It can be seen that the two needles did not interfere with obstacles during insertion. The accuracy of the needle insertion in the target will be improved by the displacement compensation caused by liver deformation.

**Fig 23:**
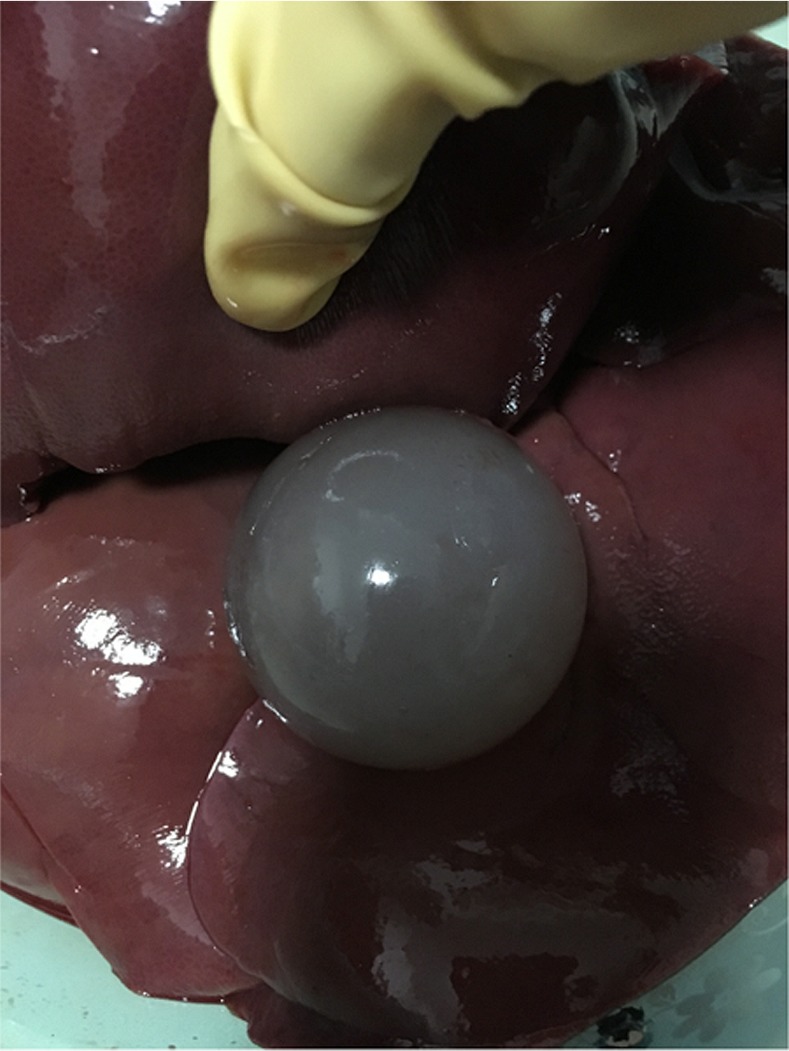
Schematic diagram of a 6-cm-diameter artificial tumor embedded in the porcine liver.

**Fig 24:**
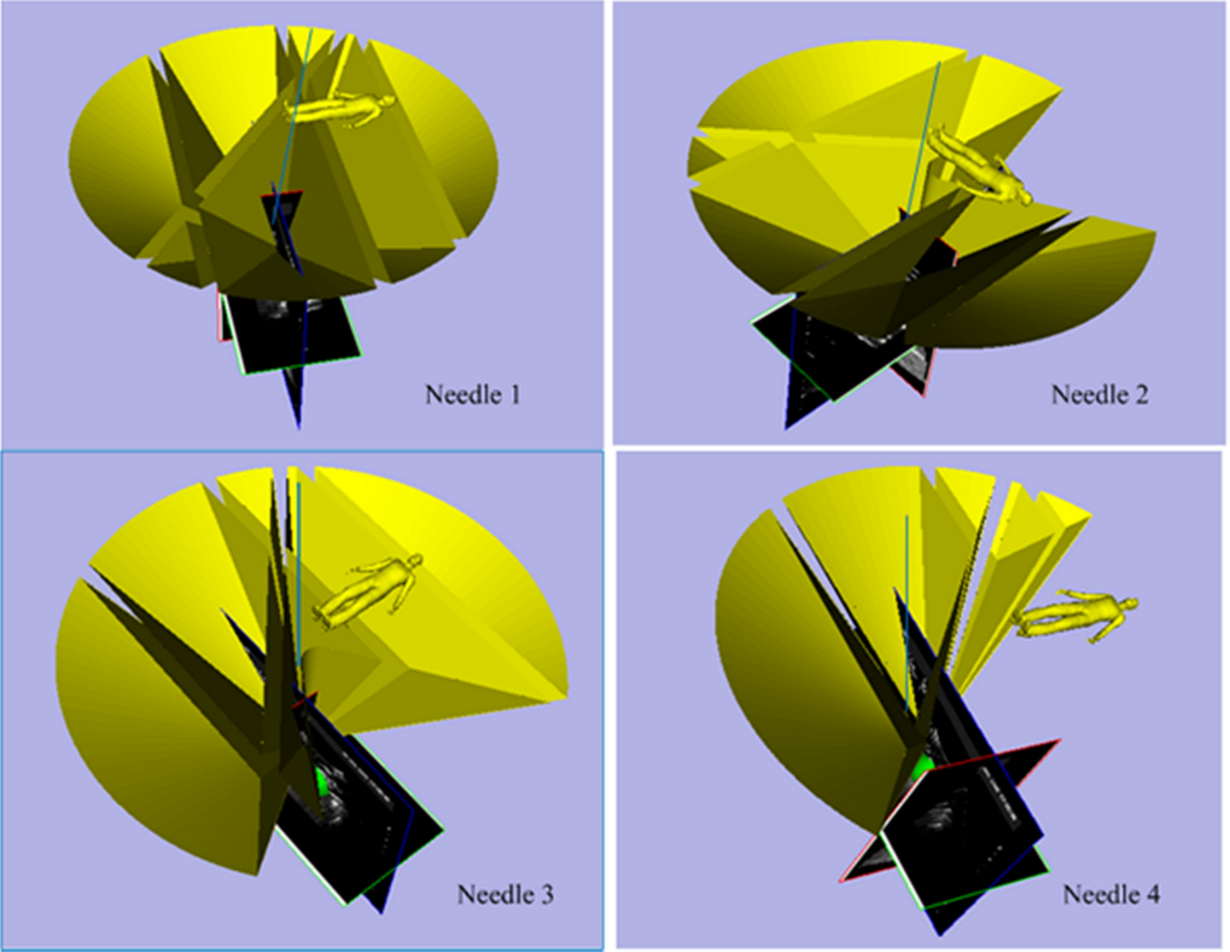
Three-dimensional scenes from the surgical navigation software for four needles surgical planning.

**Fig 25:**
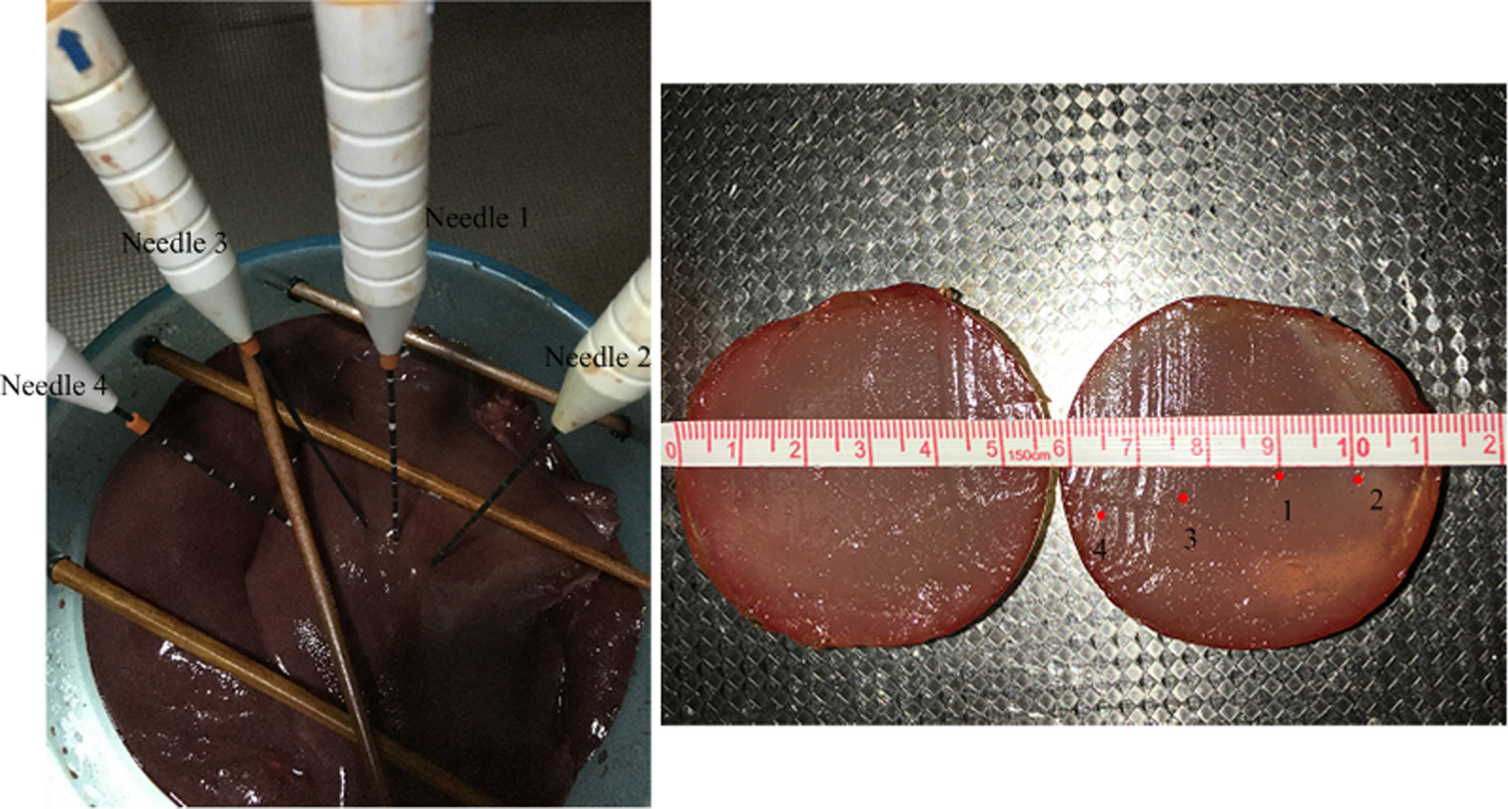
Results of the 6-cm-diameter artificial tumor experiment.

To test the proposed method still further, a 6-cm-diameter artificial tumor was embedded in the porcine liver, as shown in Fig 23. In order to facilitate the evaluation of the multiple needle insertion accuracy, four target points were chosen on the diameter of the tumor and the distances between them were the same. Repeating above steps and displacement of each target was compensated by using liver deformation analysis method. The planning results and corresponding experimental results are shown in Figs 24 and 25. We see that the needles did not interfere with each other and obstacles. We evaluated the target error by cutting the artificial tumor along the cross section which though the center of the tumor. The needle placement accuracy of the first needle is best, followed by the second needle and the third needle, the worst is the fourth needle.

We repeated the experiments 10 times by using artificial tumors with different diameter and different number of needles. 6-cm-diameter artificial tumor with 4 needles repeated 4 times; 5-cm-diameter artificial tumor with 3 needles repeated 4 times; and 3-cm-diameter artificial tumor with 2 needles repeated 2 times. The needles did not interfere with each other and obstacles in all experiments. And the target error was about 2.1 ± 0.6 mm.

## Discussion

From the above experiment, we see that the proposed multiple needle surgical planning method successfully calculated the needle CFRW and the optimal needle insertion trajectories corresponding to each target. And every needle was placed accurately by RALTCT system which achieves very high needle placement accuracy for fixed rigid target [4]. However, the accuracy will be affected by the target motion, which is inevitable in the clinical surgery. Since the insertion is monitored by 2D US imaging during the intraoperative stage in clinical surgery, the US probe was used to exert some pressure onto the patient abdomen, resulting in the deformation and motion of the liver. Also, the organ and lesion motion due to the respiratory effects will severely affect the needle placement accuracy and even cause the missing of the target in some cases [40]. Therefore, the target motion may be caused by the needle insertion, uneven pressure exerted by the US probe and the respiratory motion of the patient. Fortunately, the proposed method can be used to reduce needle placement error by target displacement compensation, as the displacement can be easily compensated into the proposed method once a reliable organ deformation model is available. Also, the target error caused by respiration can be partially compensated by the patient’s conscious regulation of breath according to the surgeon’s instruction, which was already used in clinical application of RALTCT system successfully. However, the operation of multiple needle surgery is more complicated and time-consuming. Therefore, it’s necessary to create a real-time motion compensation [40–41] model to ensure the proposed method can be used in clinical surgery.

In addition, the further clinical application of this method must consider some other prerequisites, such as the fine registration between the preoperative and intraoperative models. In order to ensure abdominal cavity environment consistency and the surgery safety, the patients should not eat any solid food after midnight prior to the day of the procedure. Then, during the surgery, the registration is carried out by body surface markers and portal vascular in the liver [42]. We also use the slice guidance method [4] to guide the surgeon adjust the position and magnitude of the pressure exerted by the US probe until the resliced image from the preoperative model matches the real-time US image, then, insert the needle.

The effectiveness of MC, radiofrequency ablation and microwave ablation in the treatment of large liver tumor has been proven by a number of clinical studies and medical practice reports [5, 43–45]. Based on existing studies, a precise and safe needle placement to hit the targets is a main factor affecting the surgery results. This was the focus of current study. Therefore, in the *in vitro* experiments, only the accuracy and safety of the planned needle insertion trajectories were verified. Our future plan is to integrate the optimal targets planning and irradiation strategy to further enhance the practicability of the proposed method in robot-assist clinical application.

## Conclusion

To our knowledge, we are the first to report the analytical expression of needle CFRW for automatic multiple needle trajectory planning in the abdomen. The clinical constraints such as the no-collision constraint, a safety margin around the target, tangency constraint, needle length constraint, and other rating factors, such as the distance to critical structures and trajectory length, are all translated into a mathematical model and implicitly expressed by non-linear equations that describe the needle CFRW. Also, the deformation of soft tissue during needle insertion and the robot’s features are considered in our planning. This method is independent of the mesh representation of related structures and the resolution of the screen used. We show that our system can automatically provide the optimal insertion trajectory for each needle in multiple overlapping coagulations and also can prevent the surgeon from manually choosing trajectories that are likely to lead to complications during the surgery. Although the analysis of deformation for soft tissue during insertion and the computation of non-linear equations that describe the needle CFRW are time-consuming, the surgical planning is preoperative, and effectiveness is more important. The results of the simulation and the *in vitro* experiment indicate the feasibility and effectiveness of this multiple-needle surgical planning. Because this is a general method, regardless of the variable operating environment, the proposed method can be readily extended to other body parts in a straightforward manner, such as for a biopsy. The accuracy of this proposed method mainly depends on the accuracy of the liver deformation model and the description of patient-specific structures. The previous research [31, 38] can ensure both abovementioned accuracies. However, to ensure the safety of the patients, during the surgery, the insertion should be always monitored by US scanning.

In future, the clinical routine will be carried out to further evaluate the proposed surgical planning. Furthermore, we intend to extend our approach to include additional objective functions, such as an intraoperative risk analysis during the insertion to increase the safety. Breathing motion will be considered in surgical planning to compensate the accuracy of surgery. Also, the extension of the operating preferences of the physician to the surgical planning involves more humanization. The idea is to compute not one but many optimal trajectories for each needle, each corresponding to a different optimal objective.
